# Magnetron sputtering of hybrid metal halide perovskites: barriers to scalable fabrication

**DOI:** 10.1039/d5el00180c

**Published:** 2026-02-09

**Authors:** Laxmi Laxmi, Vladimir V. Shilovskikh, Shivam Singh, Sneha Babu, Ronny Engelhard, Boris Rivkin, Yana Vaynzof

**Affiliations:** a Leibniz Institute for Solid State and Materials Research Dresden Helmholtzstraße 20 01069 Dresden Germany y.vaynzof@ifw-dresden.de; b Chair for Emerging Electronic Technologies, TUD Dresden University of Technology Nöthnitzer Str.61 01187 Dresden Germany

## Abstract

Radio frequency (RF) magnetron sputtering is a scalable, solvent-free, and industry-compatible deposition technique well-suited for roll-to-roll manufacturing. Despite its many advantages, this technique is not commonly used for the deposition of metal halide perovskites, which have emerged as one of the most promising photovoltaic materials. In this study, we systematically explore the barriers to depositing high-quality perovskite thin films from hybrid perovskite targets, which were prepared by pressing ball-milled dry perovskite powder. By investigating the influence of target stoichiometry, applied RF power, and Argon gas pressure on the structural, morphological, and compositional characteristics of the sputtered thin films, we identify key limitations to the application of magnetron sputtering to the fabrication of perovskite layers. Specifically, we reveal that the microstructure of these films exhibits a very rough surface and inadequate grain formation, limiting their use in subsequent optoelectronic applications. Additionally, target disintegration during sputtering poses a significant challenge, raising concerns about the feasibility of RF sputtering for roll-to-roll production of perovskite optoelectronic devices. Based on these insights, we discuss not only the limitations of the process but also potential paths to addressing these barriers.

## Abstract 2

This study examines RF magnetron sputtering of metal halide perovskites, highlighting key challenges – compositional and microstructural flaws and target disintegration – that limit its broad applicability for solar cell fabrication.

## Graphical abstract

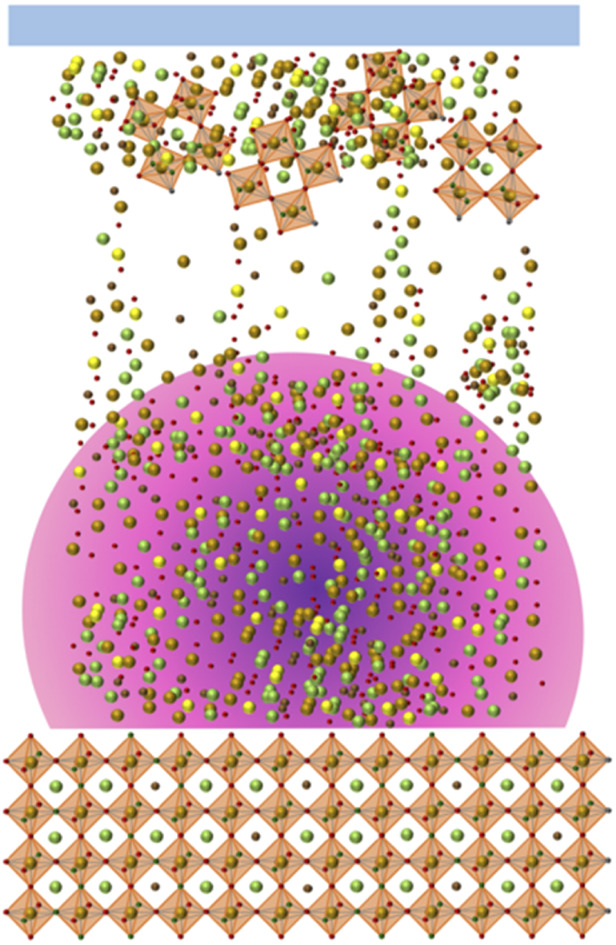

**Broader context**

Perovskite solar cells (PSCs) have rapidly emerged as the most promising next-generation photovoltaic technology, achieving efficiencies that match or surpass those of silicon-based solar cells. However, most high-performance PSCs are produced using small-scale laboratory methods, such as spin coating, which are not suitable for industrial-scale manufacturing due to their limited scalability. Therefore, developing reliable, reproducible, and large-area deposition techniques is vital to transition from laboratory research to commercial use. Magnetron sputtering, a mature and industry-standard thin-film process, offers several benefits, including excellent film uniformity, precise control of film thickness, and compatibility with large substrates. Despite these advantages, the application of this method for depositing metal halide perovskites remains relatively underexplored, with very few studies investigating its potential. Moreover, these studies often lack essential experimental details, making it difficult to reproduce the results and limiting their broader application. This work presents a systematic study of single-target magnetron sputtering for perovskite thin films, identifying key challenges such as stoichiometric control, microstructural defects, and target degradation. By identifying these issues and suggesting practical solutions, this research helps advance scalable production methods for PSCs, supporting the transition of perovskite photovoltaics from laboratory innovation to industrial manufacturing.

## 1. Introduction

Metal halide perovskites have emerged as a promising candidate for next-generation thin-film photovoltaic technology, as the power conversion efficiency (PCE) of perovskite solar cells (PSCs) has reached a record value of ∼27%.^1,2^ Despite this progress, most high-efficiency PSCs are fabricated *via* spin coating, a wet and lab-friendly technique.^3–7^ To translate the excellent photovoltaic properties of perovskites to the commercial scale, their fabrication *via* scalable deposition techniques must be developed. Since industrial-scale fabrication processes typically avoid the use of toxic solvents, dry deposition techniques are particularly attractive for upscaling perovskite-based photovoltaics.^8^ Such methods offer several advantages, including high throughput, homogeneity, material economy, safety, yield, and controllability.^9^ Reported dry deposition methods for metal halide perovskites include thermal evaporation,^10–12^ closed-space sublimation,^13^ pulsed laser deposition,^14^ electron beam evaporation,^15^ and radio frequency (RF) magnetron sputtering.^16,17^

RF magnetron sputtering is a well-established dry technique for depositing various inorganic semiconductors and insulators.^18–20^ Furthermore, it is suitable for roll-to-roll fabrication processes.^21^ Despite these advantages, the use of magnetron sputtering for depositing metal halide perovskites is uncommon. In 2016, He *et al.* reported the sputtering of metallic lead (Pb), which was then converted to methylammonium lead iodide (MAPbI_3_) perovskite through spin coating a methylammonium iodide (MAI) solution onto it.^22^ Later on, other reports focused on the sputtering of lead iodide (PbI_2_), lead oxide (PbO), or lead sulfide (PbS) on the electron transport layer. These layers were then converted to MAPbI_3_ either by dipping them in MAI solution, by MAI vapor treatment, or by methylamine (MA) vapor treatment.^23–25^ It was reported that the sputtered inorganic layer is relatively compact, requiring additional steps, such as the iodination process and dimethyl sulfoxide solvent treatment, to make it porous enough for MAI to ingress into the inorganic layer and form the perovskite phase.^23^ These additional steps, which rely on solution-based treatments, diminish the inherent advantage of the magnetron sputtering process for dry deposition.

To simplify the sputtering process, in 2018, Bonomi *et al.* reported the single-target sputtering of MAPbI_3_. To prepare the target, the perovskite powder is first synthesized *via* a mechanochemical process, then pressed into a disc shape at high pressure. The authors utilized the target to sputter at a power of 40 W and an Argon pressure of 20 µbar; however, the deposited films were not used in optoelectronic devices.^26^ Similarly, Caporali and colleagues utilized single-target sputtering of inorganic perovskites (cesium lead bromide – CsPbBr_3_ and cesium lead chloride – CsPbCl_3_) at a power of 20 W, and an Argon pressure of 2 µbar, yet no devices were fabricated using the sputtered inorganic perovskite films.^27–31^

The breakthrough for sputtered perovskites occurred in 2021, when Zhao and coworkers reported MAPbI_3_ single target sputtering, which resulted in a PSC device with a good PCE of 15%.^32^ Recently, the authors have reported photovoltaic devices with PCE of 19% and 20% for the formamidinium methylammonium lead iodide bromide mixed perovskite (FAMAPb(IBr)_3_) single target sputtering.^16,33^ Notably, to date, successful fabrication of PSC devices *via* RF magnetron sputtering has been reported only by their group. However, these studies do not explicitly disclose the applied RF power or provide details about the sputtering system employed.

Despite the promising efficiency of PSCs fabricated *via* single-target sputtering, and the method's clear potential for commercialization, a systematic investigation of deposition parameters, target composition, and their influence on film properties remains unexplored in the literature. In this work, we explored the deposition of formamidinium cesium lead iodide bromide mixed perovskite FACsPb(IBr)_3_ by RF magnetron sputtering from a single target. We selected CsFAPb(IBr)_3_ composition because it is generally considered one of the most stable compositions for perovskite solar cell fabrication.^34–37^ We systematically varied the applied power, argon flow pressure, and composition stoichiometry of the target. X-ray diffraction (XRD), X-ray photoemission spectroscopy (XPS), and microscopy techniques were employed to analyze the structural, compositional, and microstructural characteristics of the sputtered thin films. These results enable us to identify key barriers to the use of sputtering for the scalable fabrication of perovskite thin films and propose several future research directions to mitigate them.

## 2. Results and discussion

**Fig. 1:**
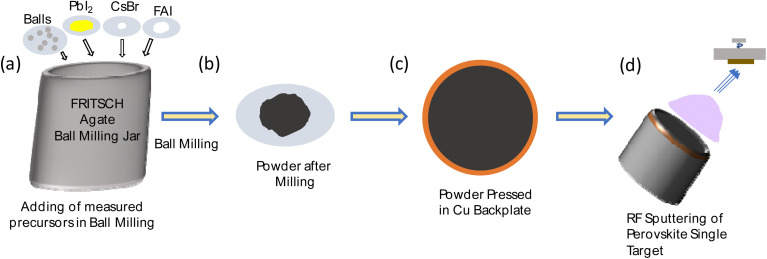
Schematic diagram of (a) pouring of measured perovskite precursors and balls in an agate jar, (b) powder obtained after ball milling, (c) target obtained after pressing of powder in Cu backplate, and (d) RF sputtering process.

Fig. 1 illustrates the synthesis and deposition process. First, we placed the balls in the jar and then added the perovskite precursors in the required ratios (see the Experimental Section for details). This was followed by the ball milling process, which was performed using a planetary ball milling machine (detailed parameters are provided in the experimental section). After completing the dry mechanochemical synthesis, the perovskite powder was pressed onto a Cu backplate using an automatic press machine to make the target. Each target was of 5 cm diameter and ∼1 mm thickness, made from 5 g of synthesized powder. The targets were utilized for RF magnetron sputtering with a range of powers and argon flow pressures as described in the following. The accessible parameter space was limited by plasma stability at low pressure and by deposition rate at low power.

### 2.1 From powder/target to thin films

#### 2.1.1 Structural and optical investigation

**Fig. 2:**
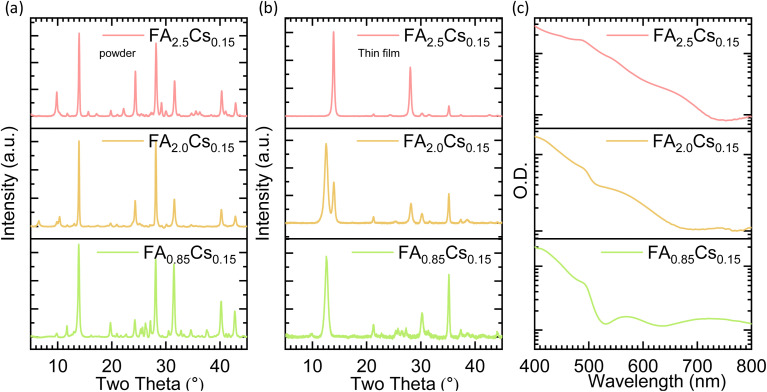
X-ray diffraction (XRD) pattern of (a) ball-milled dry powders, and (b) sputtered thin films sputtered at an applied power of 45 W and an Argon gas pressure of 20 µbar corresponding to different compositions. (c) UV-vis-NIR absorption spectra of sputtered thin films corresponding to different compositions.

Fig. 2 shows the XRD patterns of different powder compositions and the corresponding thin films derived from those powder composition-based targets. We began with a stoichiometric composition of perovskite powder with the formula of FA_0.85_Cs_0_._15_Pb(I_0_._95_Br_0.05_)_3_ (abbreviated as FA_0.85_Cs_0.15_), where we have incorporated 0.85 M FAI (1.14 g), 0.15 M CsBr (0.25 g), and 1 M PbI_2_ (3.61 g) during powder preparation to prepare a total of 5 g of powder. After the ball milling process, the powder turned black (Fig. S1). The XRD analysis of the FA_0.85_Cs_0.15_ powder revealed the formation of a perovskite phase, with prominent peaks at 2*θ* = 14° and 2*θ* = 28° (Fig. 2a). The perovskite target pressed from the FA_0.85_Cs_0.15_ powder composition was sputtered at an applied power of 45 W and an Argon gas pressure of 20 µbar, consistent with the parameters used in the initial literature report on single-target perovskite sputtering.^26^ Unexpectedly, the resulting thin film appeared yellow (Fig. S2), with XRD indicating the formation of lead iodide (PbI_2_)-rich films with a prominent peak at 2*θ* = 12.6° (Fig. 2b).^11^

We further explored the effect of sputtering conditions for the FA_0.85_Cs_0.15_ powder composition-based target by varying the applied power to 30 W and 75 W while keeping the same argon pressure of 20 µbar. At 30 W power, the results are similar to those at 45 W, as the deposited films are yellow (Fig. S3) and the XRD pattern indicates the formation of only PbI_2,_ with a prominent peak at 2*θ* = 12.6° (Fig. S3a). At 75 W power, the deposited films are slightly brown in color, and the XRD pattern shows a small peak for perovskite at 2*θ* = 14°; however, the PbI_2_ peak at 2*θ* = 12.6° still dominates. Furthermore, we have explored the variation in argon gas pressure while maintaining a constant power of 75 W. The argon gas pressure is varied to 50 µbar and 75 µbar. Again, we do not achieve the complete perovskite phase in the resulting perovskite films for these conditions (Fig. S3b), suggesting a deficiency of the organic component in the thin films. This could be due to differences in the sputtering rates of organic and inorganic components.^26^ Fine-tuning of the sputtering parameter space could, in principle, support further optimization; however, under varied conditions, we were unable to achieve perovskite phase formation using stoichiometric targets of FA_0.85_Cs_0.15_ composition.

Literature reports on other dry deposition techniques, such as thermal evaporation^38^ or pulsed laser beam deposition,^39^ suggest that a stoichiometric FAI ratio does not result in complete conversion to the perovskite phase, similar to our observations with sputtering. These methods often rely on an over-stoichiometric FAI molar ratio to facilitate the formation of the perovskite phase. Based on these reports and our observations, we increased the FAI content in the powder preparation. At first, a composition of 2.0 M FAI (1.15 M excess), 0.15 M CsBr, and 1 M PbI_2_ (FA_2.0_Cs_0.15_) was prepared. This powder is dark orange in colour (Fig. S1), with an XRD pattern confirming the formation of the perovskite phase, along with additional peaks at 2*θ* < 10° (Fig. 2a), possibly due to an excess of the FA-based lower-dimensional compound.^40^ The sputtered thin films with a 1.15 excess FAI powder-based target resulted in a light brown color, and XRD measurements revealed partial conversion to the perovskite phase, while PbI_2_ was still present in significant amounts in the films (Fig. 2b).

Subsequently, we increased FAI to 2.5 M (1.65 M excess), while maintaining 0.15 M CsBr and 1 M PbI_2_ (FA_2.5_Cs_0.15_). This powder is orange, and the XRD pattern resembles that of 1.15 M excess powder with a more pronounced peak at 2*θ* < 10° (Fig. 2a), possibly due to an even greater excess of FA-based low-dimensional compound.^40^ The sputtered thin films with 1.65 excess FAI powder-based target resulted in a dark brown color, and the XRD revealed a seemingly complete conversion to the perovskite phase (Fig. 2b).

Fig. 2c presents the UV-vis-NIR absorption spectra of sputtered films based on different compositions. For the FA_0.85_Cs_0.15_ composition, the film shows dominant absorption below 520 nm. With increasing FAI content in the FA_2.0_Cs_0.15_ and FA_2.5_Cs_0.15_ compositions, the absorption onset red shifts, accompanied by increased absorption in the visible region. The absorption onset for the FA_2.0_Cs_0.15_ and FA_2.5_Cs_0.15_ target compositions is at 640 nm and 710 nm, respectively. These results are consistent with the formation of a perovskite phase using the largely overstoichiometric targets. To complement the absorption measurements, we also performed photoluminescence (PL) spectroscopy characterization (Fig. S4). We observe that the FA_0.85_Cs_0.15_ shows nearly no emission, while the spectrum of the FA_2.0_Cs_0.15_ sample reveals an emission peak at 520 nm, associated with PbI_2_ (ref. 41) as well as broad emission at lower energies originating from the perovskite. The FA_2.5_Cs_0.15_ composition shows only perovskite-phase emission, in agreement with the XRD results.

To investigate whether these observations are related to the presence of organic cations in the perovskite, we have also explored the inorganic composition CsPbI_2_Br. We investigated the stoichiometric CsPbI_2_Br composition (Cs_1.0_), CsPbI_2_Br with 0.5 M excess CsBr (Cs_1.5_), and CsPbI_2_Br with 1.0 M excess CsBr (Cs_2.0_). Similar to the case of hybrid perovskites, we could achieve perovskite phase formation in inorganic perovskites only under harsh sputtering conditions (75 W, 75 µbar) for CsPbI_2_Br (Cs_1.0_) or with an excess 1 M CsBr (Cs_2.0_) composition (Fig. S5–S7).

#### 2.1.2 Compositional investigation

**Fig. 3:**
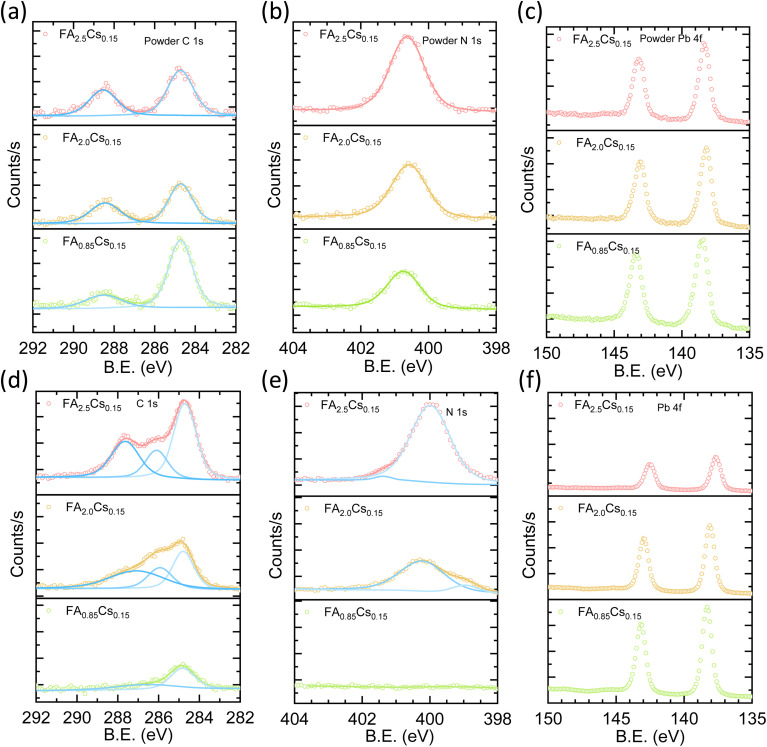
(a) C 1s, (b) N 1s, and (c) Pb 4f core level spectra in different compositions of powders. (d) C 1s, (e) N 1s and (f) Pb 4f core level spectra in sputtered thin films corresponding to different powder compositions.

To investigate the discrepancy in the phase formation between powders and sputtered thin films, we analyzed their chemical compositions using XPS measurements. Fig. 3a represents the C 1s core level spectra of FA_0.85_Cs_0.15_, FA_2.0_Cs_0.15_, and FA_2.5_Cs_0.15_ powders. The peak at 284.8 eV is attributed to adventitious carbon (C–C) and is used for charge correction for all other core-level spectra. The peak at 288.4 eV can be assigned to CH(NH_2_)_2_^+^ (FA^+^)^42^ carbon species. Similar to the C 1s spectra, the N 1s spectra also show a characteristic peak of FA at 400.5 eV for all three powders, as shown in Fig. 3b. Fig. S8 shows the relative atomic % of elements with respect to Pb. Although powders were mixed in a specific ratio, elemental quantification always shows a higher atomic % of C and N compared to Pb than the intended ratio. We attribute the higher C/Pb ratio primarily to surface contamination, as the composition was determined by XPS, which is inherently surface-sensitive. Adventitious carbon and surface oxidation can therefore lead to an apparent deviation of the surface from the bulk stoichiometry, which, as the XRD patterns already show, indicates a perovskite phase. However, in the case of sputtered thin films based on FA_0.85_Cs_0.15_ composition, we observed only a small peak for the FA-related C 1s and a negligible signal for N 1s spectra, as shown in Fig. 3d and e, respectively. Quantitative evaluation of the elemental composition from the XPS data of FA_0.85_Cs_0.15_ thin films reveals a significant deficiency of C (C/Pb ratio of 0.35, expected ∼0.85) and N (N/Pb ratio of 0.12, expected ∼1.7). The XPS spectra of Pb 4f, Br 3d, I 3d, and Cs 3d exhibit a single doublet for both powder and thin films (Fig. 3c, f and S9). The XPS spectra of sputtered thin films of stoichiometric compositions at other sputtering parameters of power and pressure variations show a broad peak with a long tail for C 1s, and N 1s than the XPS spectra of these elements in the powder (Fig. S10 and S11), suggesting a partial decomposition of the organic compounds during sputtering. As expected, increasing the amount of excess FAI increases the atomic % of C and N relative to Pb in the XPS signal for both powders and films (Fig. S8). However, the XPS of sputtered thin films consistently shows lower C and N atomic percentages than their powder counterparts (Fig. S8). Additionally, the C 1s core level spectra of sputtered thin films display a significant additional peak around 286.5 eV, which is not observed in the powder XPS signal. We suspect this peak emergence could be due to the degradation of the FA^+^ ion during the sputtering process.^43^ Adding extra FAI to compensate for the reduced C and N atomic percentages significantly raises the I/Pb atomic ratio (4.75) in FA_2.5_Cs_0.15_ composition-based thin films.

Taken together, these results indicate an insufficient transfer of the stoichiometric target composition to the final sputtered films, with a notable absence of the organic components. While increasing the FAI amount in the target composition leads to an improved perovskite formation, compositional analysis reveals that this is associated with the introduction of additional impurities (most likely due to a decomposition of FAI during sputtering) and a significant excess of I, both of which reduce the optoelectronic quality of the deposited films.

### 2.2 Microstructure of thin films

**Fig. 4:**
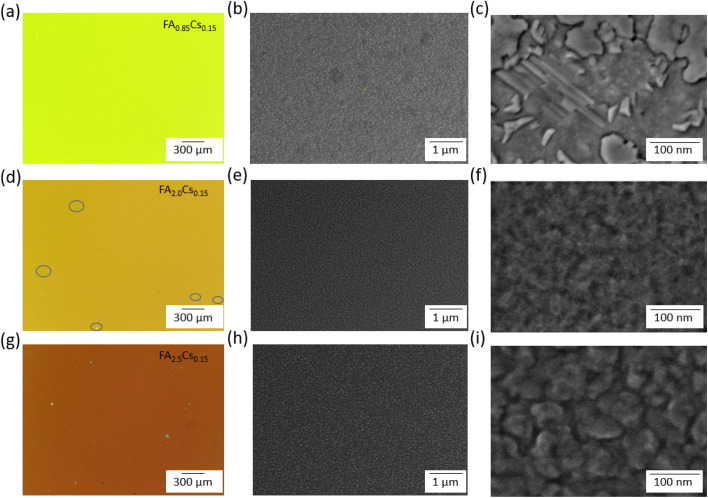
Optical and scanning electron microscopy (SEM) images of (a)–(c) FA_0.85_Cs_0.15_, (d)–(f) FA_2.0_Cs_0.15_, and (g)–(i) FA_2.5_Cs_0.15_ composition-based sputtered thin films. Microstructural flaws in FA_2.0_Cs_0.15_ are marked in circles.

The microstructure of sputtered thin films was examined using optical microscopy and scanning electron microscopy (SEM) (Fig. 4). Cross-sectional SEM images show that the deposited films have a thickness of ∼200 nm, independent of composition (Fig. S12). Optical images at 100× magnification of the FA_0.85_Cs_0.15_ composition-based film, deposited at 45 W and 20 µbar, show a clean and smooth surface, as depicted in Fig. 4a. The same applies to some other sets of sputtering parameters (30 W, 20 µbar, 45 W, 20 µbar, 75 W, 50 µbar, and 75 W, 75 µbar) (Fig. S13 and S14). However, the FA_0.85_Cs_0.15_ composition-based film deposited at 75 W and 20 µbar shows a surface with many macroscopic particles, and the same is observed for both FAI-rich composition-based films' surfaces in the optical microscope at 100× magnification (Fig. 4e, g and S13). Particle concentration on the film surface seems to be proportional to the FAI excess in the composition. SEM images reveal a compact film surface for all three compositions; however, for the FA_0.85_Cs_0.15_ composition-based film, with all different sets of sputtering parameters, lead iodide flakes are observed on the film surface (Fig. 4a, c, S8 and S13).^44,45^ In contrast, FA-rich compositions have a completely different surface. SEM images of the FA_2.0_Cs_0.15_ composition-based film does not show the typical polycrystalline structure of perovskite thin films (*i.e.*, the presence of clearly identifiable perovskite grains/domains). In the case of the FA_2.5_Cs_0.15_ composition-based film, the structure begins to resemble the formation of grains (Fig. 4f and i), albeit with sizes of approximately 50 nm. Poor grain-containing microstructure at the nanoscale and the presence of macroscopic sputtering artifacts limit the applicability of the sputtered films for further optoelectronic device applications, despite the promising crystallinity indicated by the XRD pattern. Poor grain microstructure is not limited to hybrid FACs perovskite; we have also observed similar microstructure in inorganic perovskites with different compositions and sputtering deposition parameters (Fig. S15–S17).

### 2.3 Stability of targets upon sputtering

**Fig. 5:**
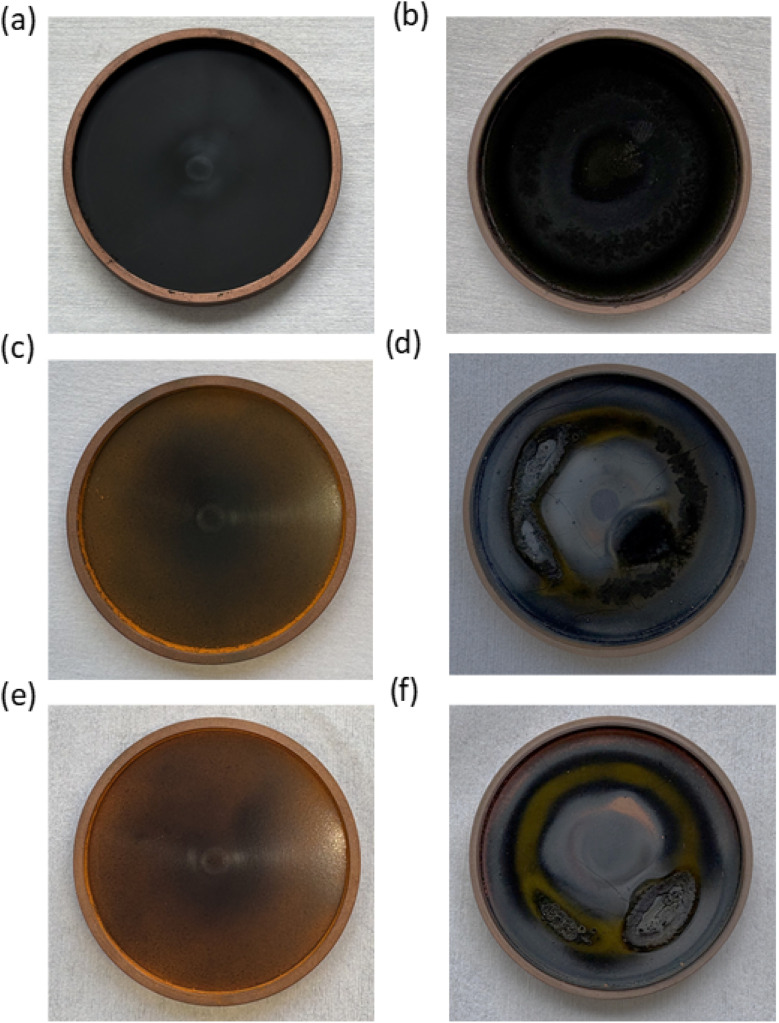
Pictures of target with different compositions (a) & (b) FA_0.85_Cs_0.15_, (c) & (d) FA_2.0_Cs_0.15_, and (e) and (f) FA_2.5_Cs_0.15_, left panel (before sputtering) and right panel (after sputtering).

Since the sputtering process has the potential for roll-to-roll fabrication, target stability is a crucial factor that has not been discussed in the literature until now. We note that roll-to-roll fabrication targets typically have different geometries, such as long rectangular plates or cylindrical tubes, which differ from those used in our study. However, this difference does not diminish the relevance of concerns about target breakage, which can occur during the first use of the target. Fig. 5 displays the appearance of the targets before and after sputtering for each of the tested compositions.

The target with a FA_0.85_Cs_0.15_ composition appeared black before sputtering, and after sputtering, it seems darker but intact (Fig. 5a and b). While this target may be suitable for reuse, it does not lead to the formation of perovskite phases on the substrates. Targets with FAI-rich compositions have a slightly orange color before sputtering, due to the powders' orange color (Fig. 5c and e). After sputtering, both FAI-rich compositions exhibit severe damage to the targets, characterized by cracks and holes. In addition to damage to the over-stoichiometric target compositions, we also observe damage to the stoichiometric target under harsh sputtering conditions, *i.e.*, higher applied power (75 W) and high argon flow pressure (75 µbar) (Fig. S18–S20). Inorganic perovskite composition-based targets are even more sensitive to sputter deposition: under all processing conditions, target breakage occurred (Fig. S21–S23). We have observed that key deposition parameters, specifically the RF power, argon pressure, and instrument details, were not reported in earlier work.^32^ Our results indicate that target disintegration during sputtering raises concerns about process reproducibility and presents challenges to the application of sputtering for a scalable fabrication of perovskite layers. Considering that sputtering of halide perovskites is still far less explored than other deposition methods, comprehensive reporting of deposition conditions and chamber configurations is essential to enable reproducibility within the community.

## 3. Barriers to sputtering of metal halide perovskites and future research directions

Our observations and results lead to the following conclusions regarding the single-target RF magnetron sputtering of hybrid metal halide perovskites (MHPs). Structural studies reveal that gentle sputtering yields organic-deficient films in which no perovskite phase forms. Perovskite phase formation in sputtered films is observed only with over-stoichiometric target compositions (Fig. 2). In such cases, the films exhibit a significant excess of iodine and unidentified organic components, likely associated with degraded FAI (Fig. 3). Our microscopy results indicate that films that showed either partial or complete conversion to the perovskite phase always exhibited flawed microstructure on multiple scales. On the nanoscale, the grain-like features we observe are approximately 50 nm in size, resulting in a high density of grain boundaries, which are typically associated with defects. On the macroscale, the films are covered with large particles (tens of microns in size), rendering them incompatible with thin-film technologies (Fig. 4). Specifically, these compositional and microstructural limitations in sputtered perovskite thin films prevent their use in functional perovskite solar cells.

Another key observation concerns the longevity of the sputtering targets. Targets crack easily during the first use upon sputtering, even with moderate applied power conditions (Fig. S24), especially when using over-stoichiometric compositions of the target (Fig. 5). This not only makes the deposition uncontrollable but also prevents the targets from being reused, thereby increasing material consumption. While very gentle sputtering conditions do not lead to apparent target destruction, they are not suitable for perovskite deposition, as no perovskite phase forms.

To address these barriers, we propose several future research directions that may help mitigate them. Existing sputter chamber architectures are designed for the deposition of standard materials, such as inorganic semiconductors or metals. Sputter deposition of perovskites may require highly specialized equipment, including adjustable sample-target distances, adequate target cooling, freely adjustable RF/DC power, and electrical stimulation. Here, the field could draw inspiration from advancements in the design of thermal evaporation chambers for perovskites, where specially designed shields and temperature-controlled chamber walls have been shown to improve the quality of the evaporated layers. To understand the growth mechanisms underlying the formation of sputtered perovskite layers, *in situ* characterization using optical or X-ray methods can be very helpful. In particular, sputtering the target may heat it, triggering two processes in parallel: sputtering and thermal vapor deposition. The significant differences in the properties of the organic and inorganic components in the target may lead to different deposition rates for the two processes. *In situ* characterization of the target and the deposited film may shed light on the mechanisms involved, thus allowing the development of methods to deposit high-quality films by magnetron sputtering. Our results suggest that single-target sputtering is a challenging process that might not be suitable for the deposition of metal halide perovskites at scale. Alternative methods, such as co-sputtering from multiple targets or sequential sputtering, may be more effective. Significantly, such methods can not only enhance control over the deposition of each component but also enable the reuse of targets for multiple depositions. Finally, sputter deposition of MHP is an application-driven field; community members should feel encouraged to openly discuss current challenges, including yield and reproducibility, exact preparation conditions of target material, and target reuse.

## 4. Experimental section

### 4.1. Materials

Formamidinium iodide (FAI) was purchased from Greatcell solar. Cesium bromide (CsBr) and lead iodide (PbI_2_) were purchased from TCI chemicals. All the materials were used as received.

### 4.2. Powder preparation

A total of 5 g of powder was weighed into each jar, and before filling the jars with powder, 14 balls per gram were added to each jar. The stoichiometric powder for hybrid perovskite was prepared by weighing 0.85 M FAI (1.143 g), 0.15 CsBr (0.249 g), and 1 M PbI_2_ (3.606 g) in an agate jar. For the preparation of inorganic perovskite powder, we used 1 M CsBr (1.89 g) and 1 M PbBr_2_ (4.10 g). The precursors were weighed inside a nitrogen glove box, and the jars were closed within the glove box. Afterwards, the jars were placed in a FRTISCH planetary ball milling machine. The ball milling parameters were 800 rpm for 30 minutes in 4 cycles, with a 10 minutes pause after each cycle. Afterward, the jars were returned to the glove box, and the black powder was collected into a clean vial. As described in the main text, an excess amount of FAI was added for non-stoichiometric compositions.

### 4.3. Target preparation

A copper (Cu) back plate with a 5 cm diameter was filled with 5 g of powder, which was leveled and then covered with the pressing stamp. The powder in the Cu back plate was pressed in an automatic press machine under atmospheric conditions at 95 kN for 30 minutes.

### 4.4. Thin film deposition and characterization

The target in the Cu back plates was installed in the benchtop RF magnetron sputtering chamber from NanoPVD (Fig. S25). The sputtering chamber used for deposition is dedicated to perovskite sputtering. Frequent baking and cleaning of the chamber were performed to prevent moisture or organic contamination. To do a systematic study of sputtering deposition parameters, we have followed the same procedure for powder and target preparation. The targets' density is approximately 3 g cm^−3.^ Cleaned ITO substrates of 12 mm × 12 mm size were kept on the substrate holder at a distance of ∼10 cm from the target. After evacuating the chamber to a pressure of 1 × 10^−5^ mbar, argon gas was released at a flow rate of 20 µbar. The applied RF power was 45 W, which was ramped in 7.5 W steps. The deposition rate was monitored using a quartz crystal monitor. Before each deposition on substrates, we performed pre-sputtering until achieving a stable deposition rate, which typically required only ∼5 minutes. The deposition rate depends on applied RF power, argon pressure, and target composition. The deposition was performed at room temperature. The sputtering system is not connected to the glovebox. After venting the sputtering system with dry nitrogen, we immediately transfer the samples into the glovebox to minimize exposure to air and humidity, typically for less than 2 minutes.

The sputtered films were post-annealed at 150 °C for 2 minutes for hybrid composition and at 200 °C for 2 minutes for inorganic composition on preheated hotplate inside the nitrogen glove box. For each deposition, we deposited a thickness of 200 nm, which was confirmed using a Dektek profilometer.

X-ray diffraction was measured with Cu Kα (*λ* = 1.54 Å) radiation and a high-energy resolution HyPix-3000 detector in a Smart Lab Rigaku X-ray diffractometer in ambient conditions. The scans (theta-2theta mode, 2*θ* = 5°–40°, step size 0.02°, 20° min^−1^, and 8.1 kW source power) were measured in Parallel Beam Geometry. To record the ultraviolet-visible (UV-vis) absorbance spectra of thin films, we used a Shimadzu UV-3100 spectrometer in air. Steady-state PL was measured using a PicoQuant spectrometer equipped with a 405 nm laser. For XPS measurements, we used an XR6 monochromated Al Kα source (*hυ* = 1486.6 eV) with a 650 µm spot size. We used pass energies of 100 and 20 eV for the survey and core-level spectra, respectively. We normalized and calibrated the spectra according to the C1s peak at 284.8 eV and analyzed the data with Thermo Scientific's Avantage software.

Using a Keyence digital microscope, we examined the morphology of thin films at lower magnification (100×). Scanning electron microscopy was performed using Apreo (Thermo Scientific, USA) at 2 keV accelerating voltage and 50 pA beam current to study the morphology of thin films at higher magnification without introduction of visible beam damage (10kX and 100k).

## 5. Conclusions

In this work, we systematically explored the influence of target stoichiometry, applied RF power, and Argon gas pressure on the structural, morphological, and compositional characteristics of the sputtered thin films. We identified several barriers to the application of single-target sputtering to metal halide perovskites, including incomplete perovskite phase formation, nano- and macroscale structural flaws, and severe target degradation. These challenges must be addressed before sputtering becomes suitable for the large-scale deposition of perovskites. We outline potential research directions that can help resolve these challenges.

## Conflicts of interest

The authors declare no conflict of interest.

## Supplementary Material

EL-002-D5EL00180C-s001

## Data Availability

The data supporting this study's findings are available from the corresponding author upon reasonable request. Supplementary information (SI): This contains Fig. S1–S25. See DOI: https://doi.org/10.1039/d5el00180c.
