# Supplementary material for: Magnetron sputtering of hybrid metal halide perovskites: barriers to scalable fabrication
Source: EES Solar. 2026 Feb 9;2(2):469–78. doi: 10.1039/d5el00180c (PMC12927458; doi:10.1039/d5el00180c)
Supplement: EL-002-D5EL00180C-s001 [file EL-002-D5EL00180C-s001.pdf]

Supplementary Information (SI) for EES Solar.
This journal is © The Royal Society of Chemistry 2026

Magnetron Sputtering of Hybrid Metal Halide Perovskites: Barriers to Scalable Fabrication

Laxmi Laxmi, Vladimir V. Shilovskikh, Shivam Singh, Sneha Babu, Ronny Engelhard, Boris Rivkin, and Yana Vaynzof*

Leibniz-Institute for Solid State and Materials Research Dresden, Helmholtzstraße 20, 01069 Dresden, Germany

Chair for Emerging Electronic Technologies, Technical University of Dresden, Nöthnitzer Str.61, 01187 Dresden, Germany

Corresponding Author

*Yana Vaynzof, e-mail: y.vaynzof@ifw-dresden.de

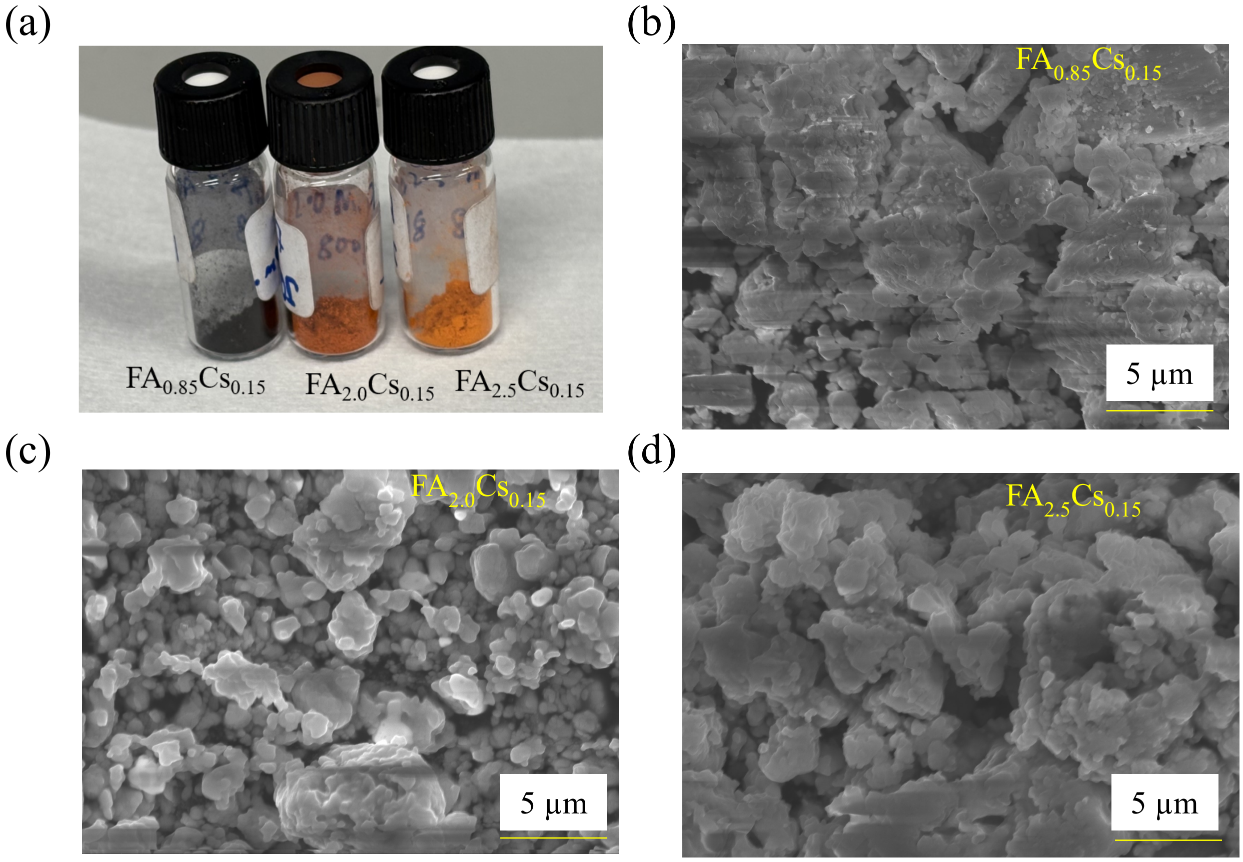

Figure S1. (a) Prepared powders with different compositions. SEM images of powders with different compositions (b) $FA_{0.85}Cs_{0.15}$, (c) $FA_{2.0}Cs_{0.15}$ and (d) $FA_{2.5}Cs_{0.15}$.

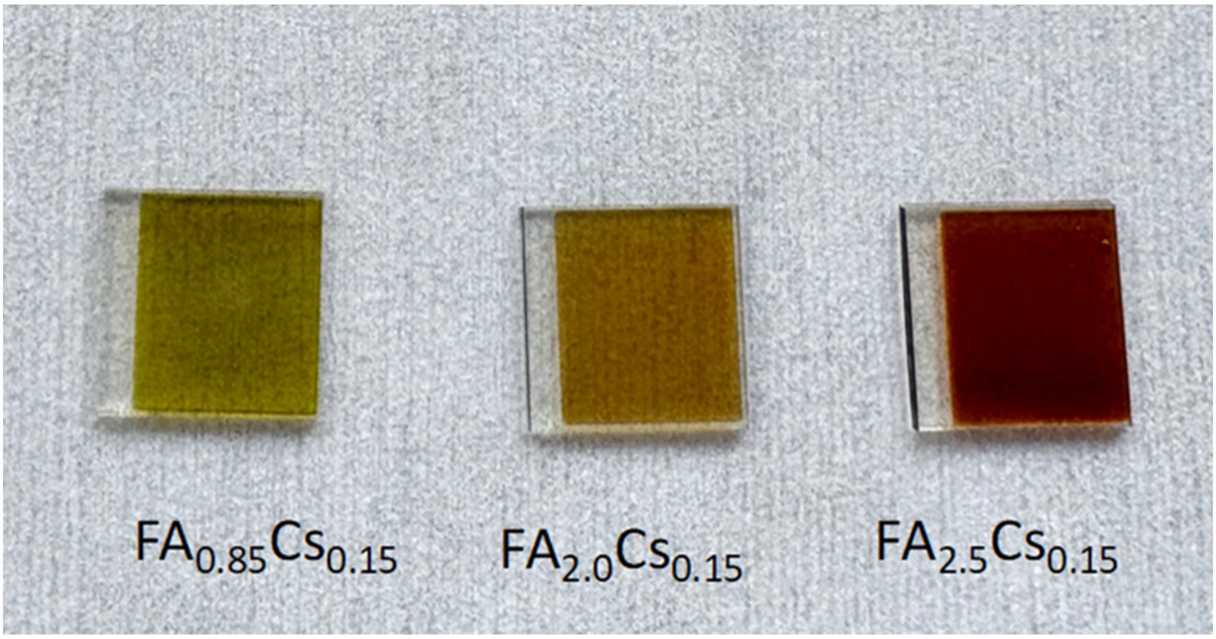

Figure S2. Images of sputtered thin films with different compositions (a) $FA_{0.85}Cs_{0.15}$, (b) $FA_{2.0}Cs_{0.15}$ and (c) $FA_{2.5}Cs_{0.15}$.

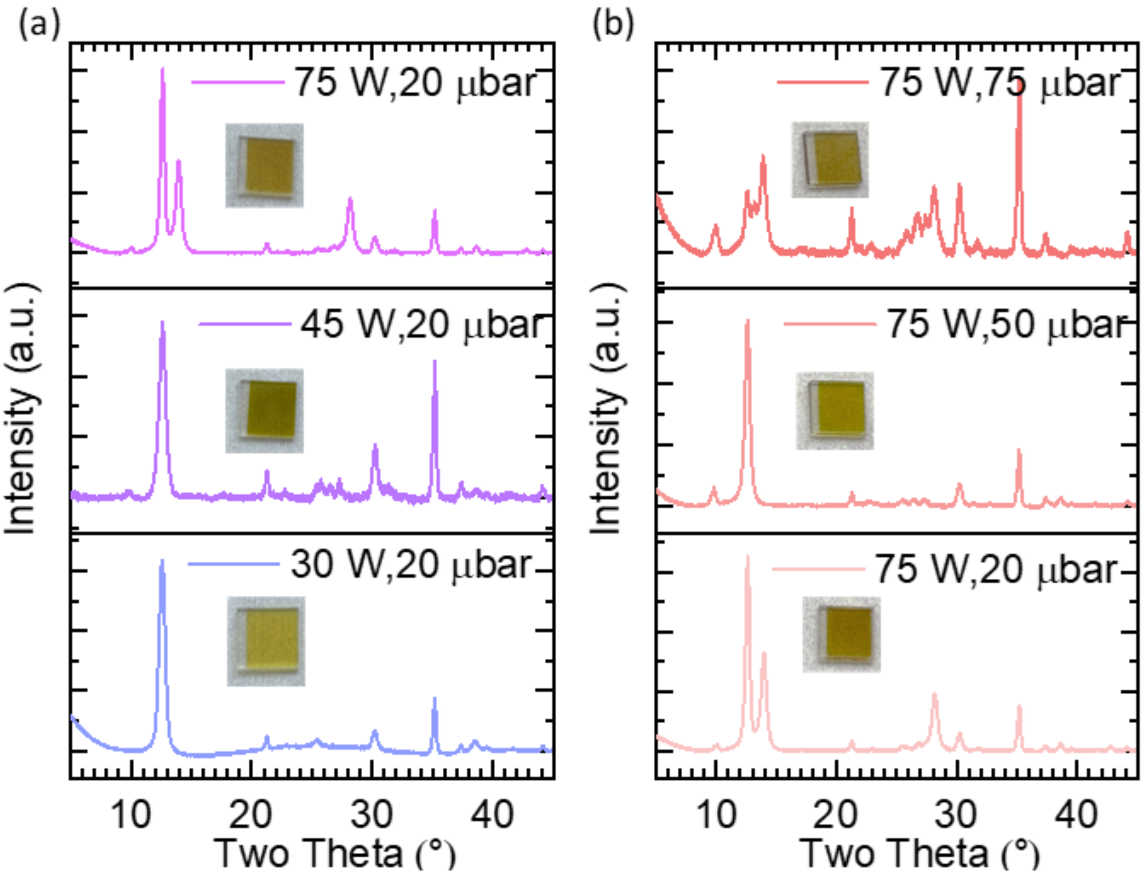

Figure S3. X-ray diffraction (XRD) pattern of sputtered thin films at (a) different applied power, and (b) different argon gas pressure for $FA_{0.85}Cs_{0.15}$ composition. Inset of (a) and (b) shows the images of sputtered thin films at the mentioned sputtering process parameters.

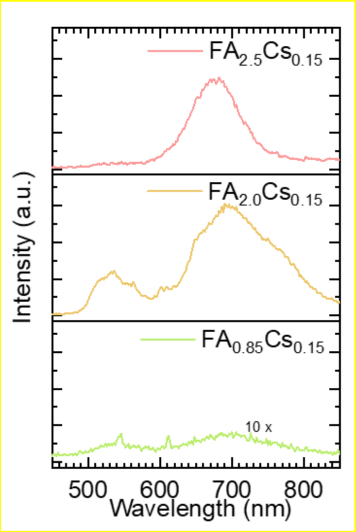

Figure S4. PL emission spectrum of films with different compositions.

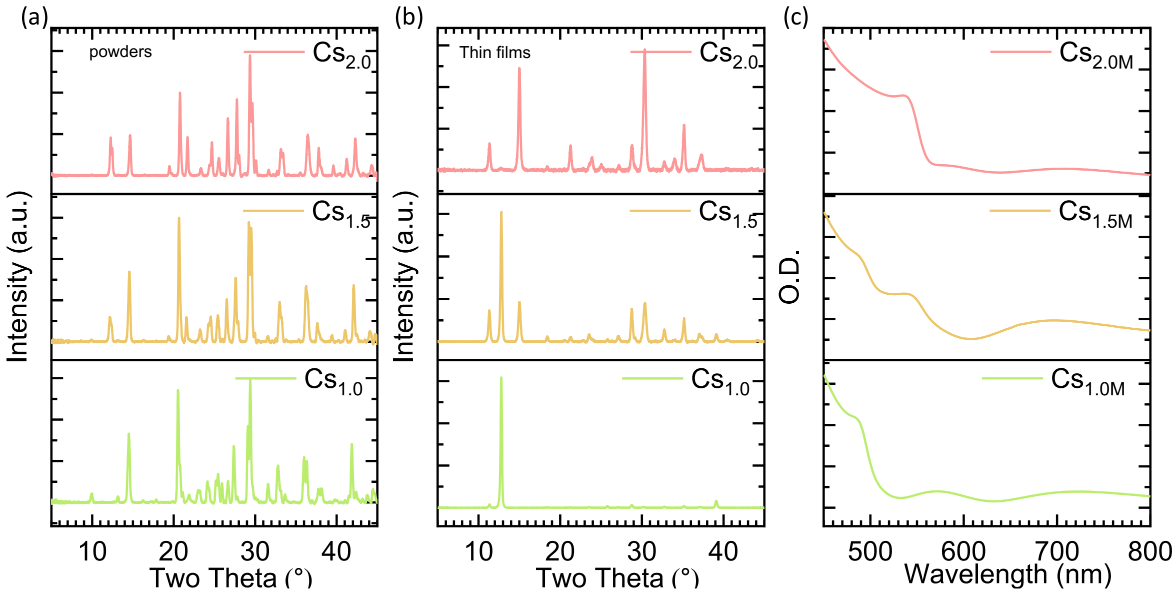

Figure S5. X-ray diffraction (XRD) pattern of (a) ball-milled dry powders, and (b) sputtered thin films sputtered at an applied power of 45 W and an Argon gas pressure of 20 µbar corresponding to different compositions. (c) UV-vis-NIR absorption spectra of sputtered thin films corresponding to different compositions.

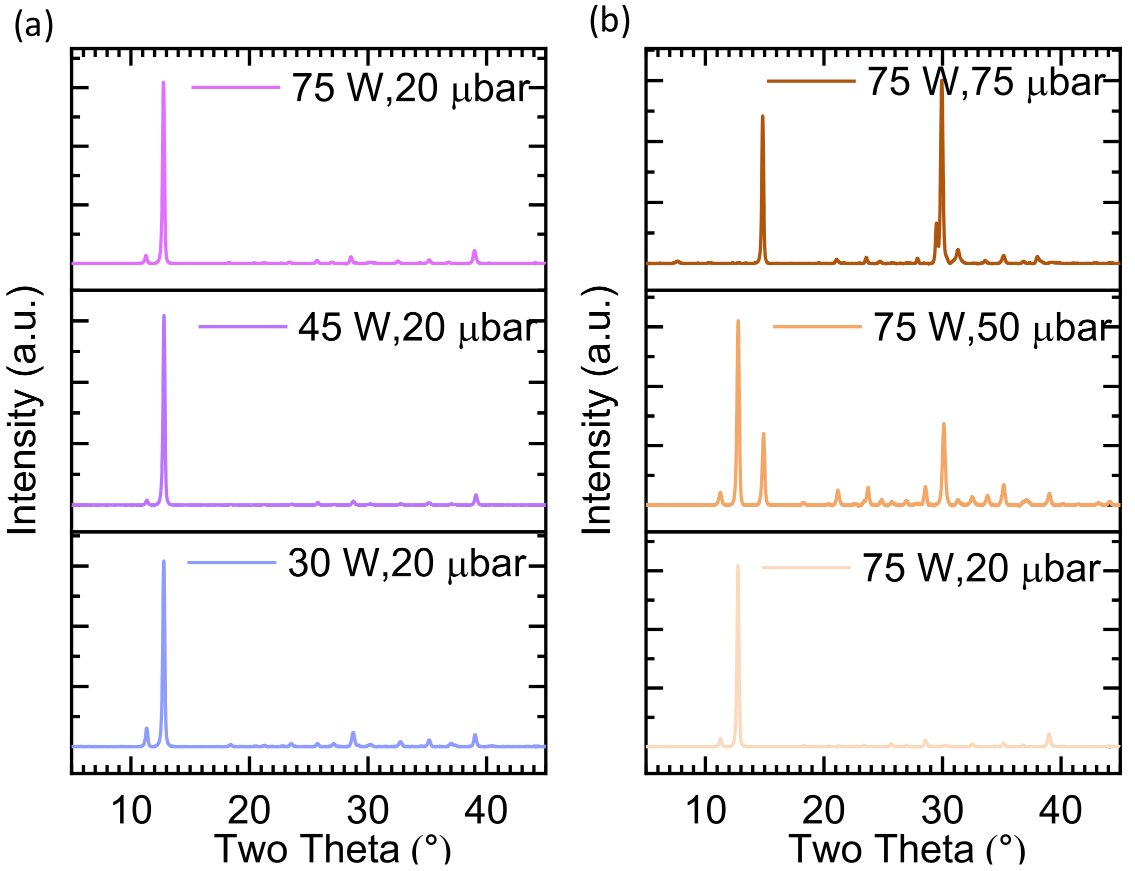

Figure S6. X-ray diffraction (XRD) pattern of sputtered thin films at (a) different applied power, and (b) different argon gas pressure for $Cs_{1.0}$ composition.

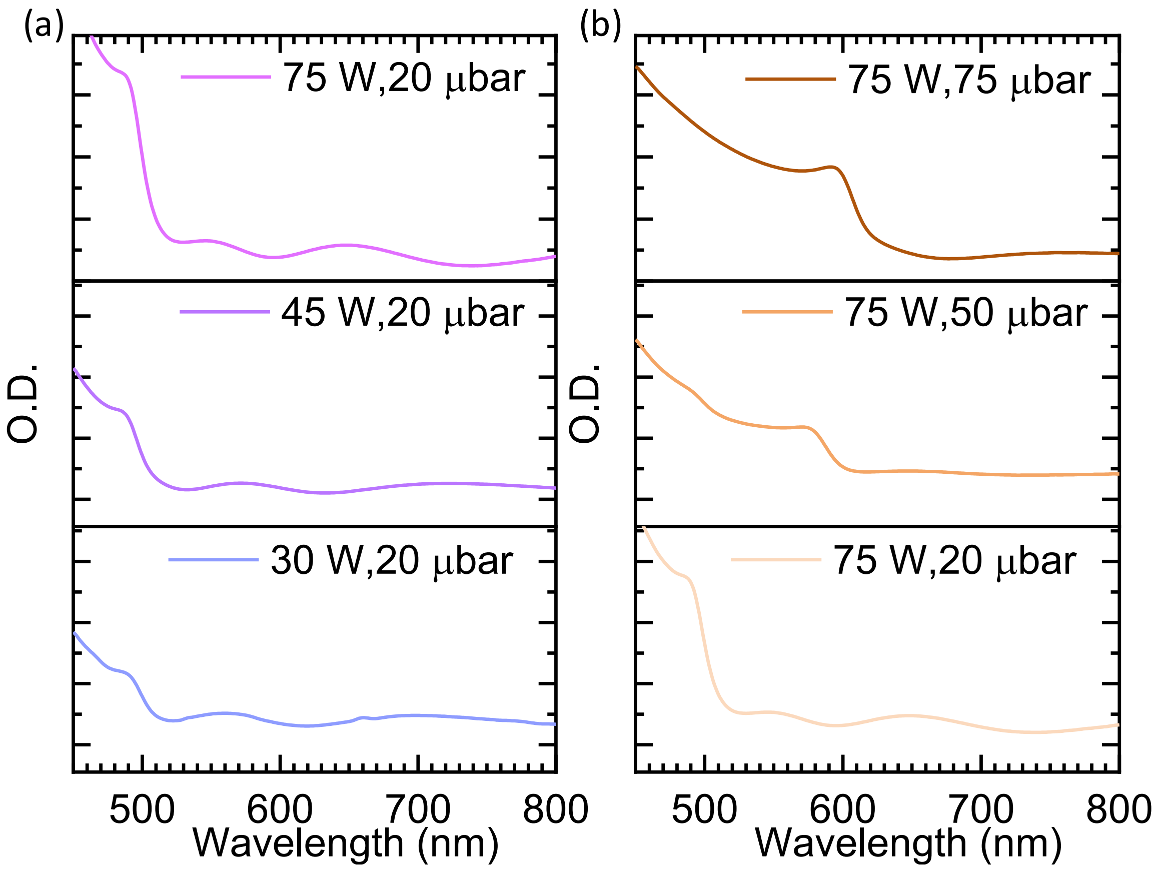

Figure S7. Absorption spectrum of sputtered thin films at (a) different applied power, and (b) different argon gas pressure for $Cs_{1.0}$ composition.

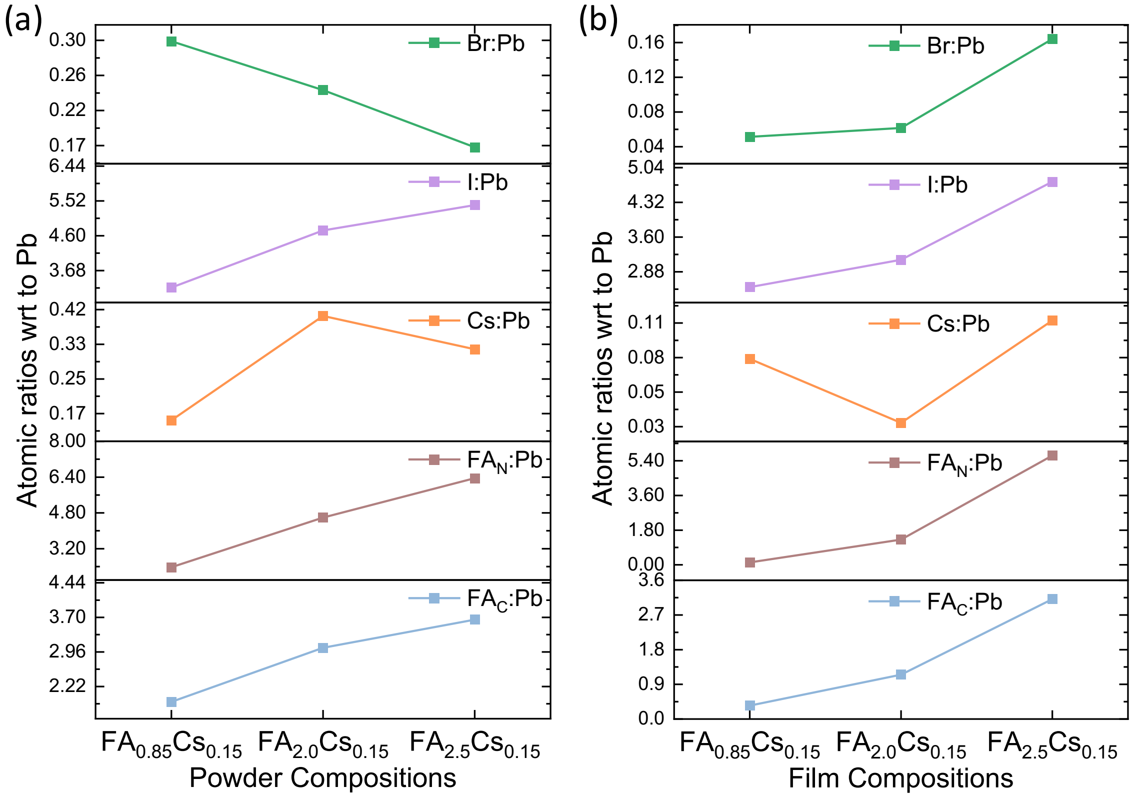

Figure S8. Element ratios with respect to Pb in (a) powders and (b) films with different compositions.

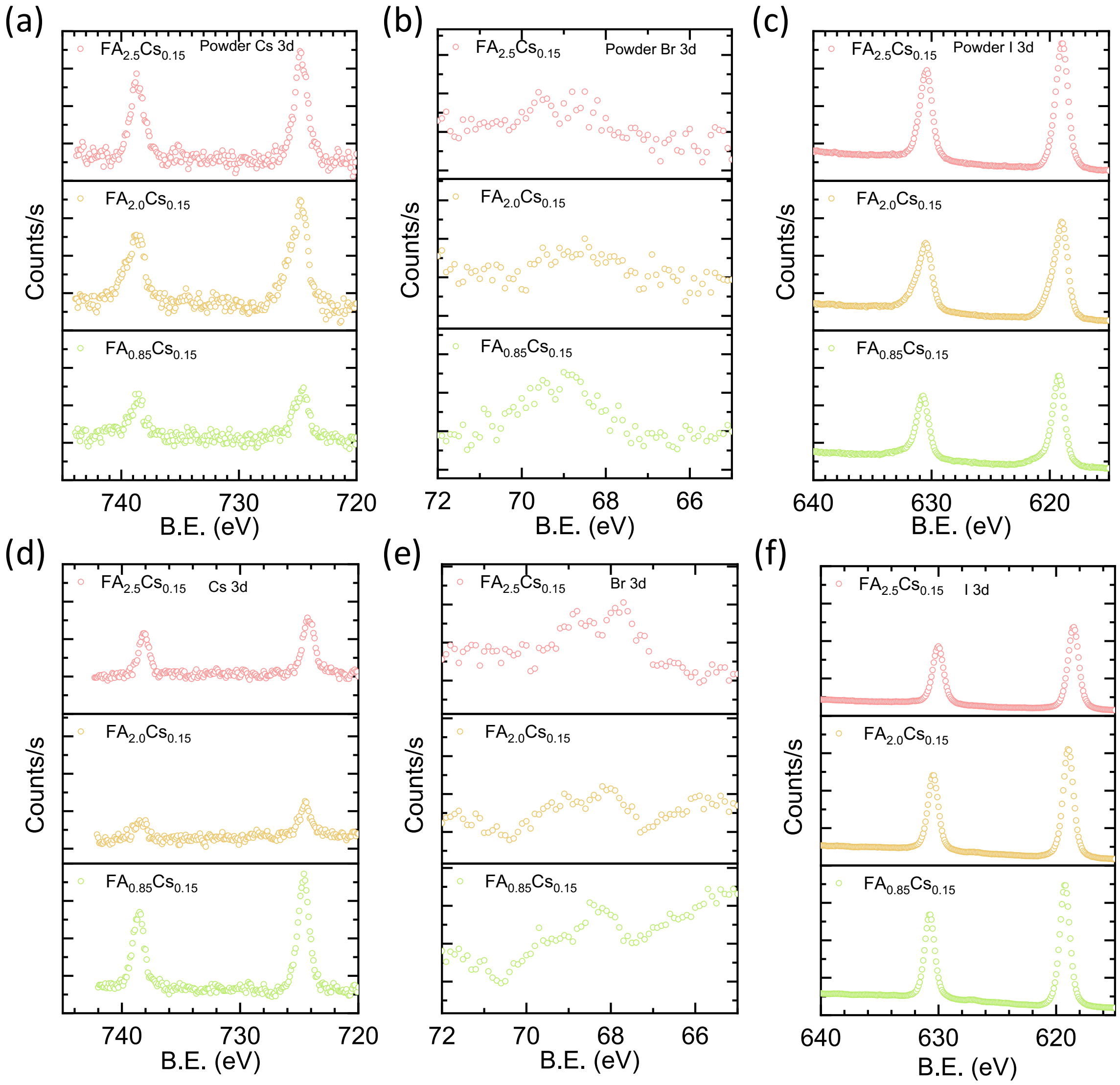

Figure S9. (a) Cs 3d, (b) Br 3d, and (c) I 3d core level spectra in different compositions of powders. (d) Cs 3d, (e) Br 3d and (c) I 3d core level spectra in sputtered thin films corresponding to different powder compositions.

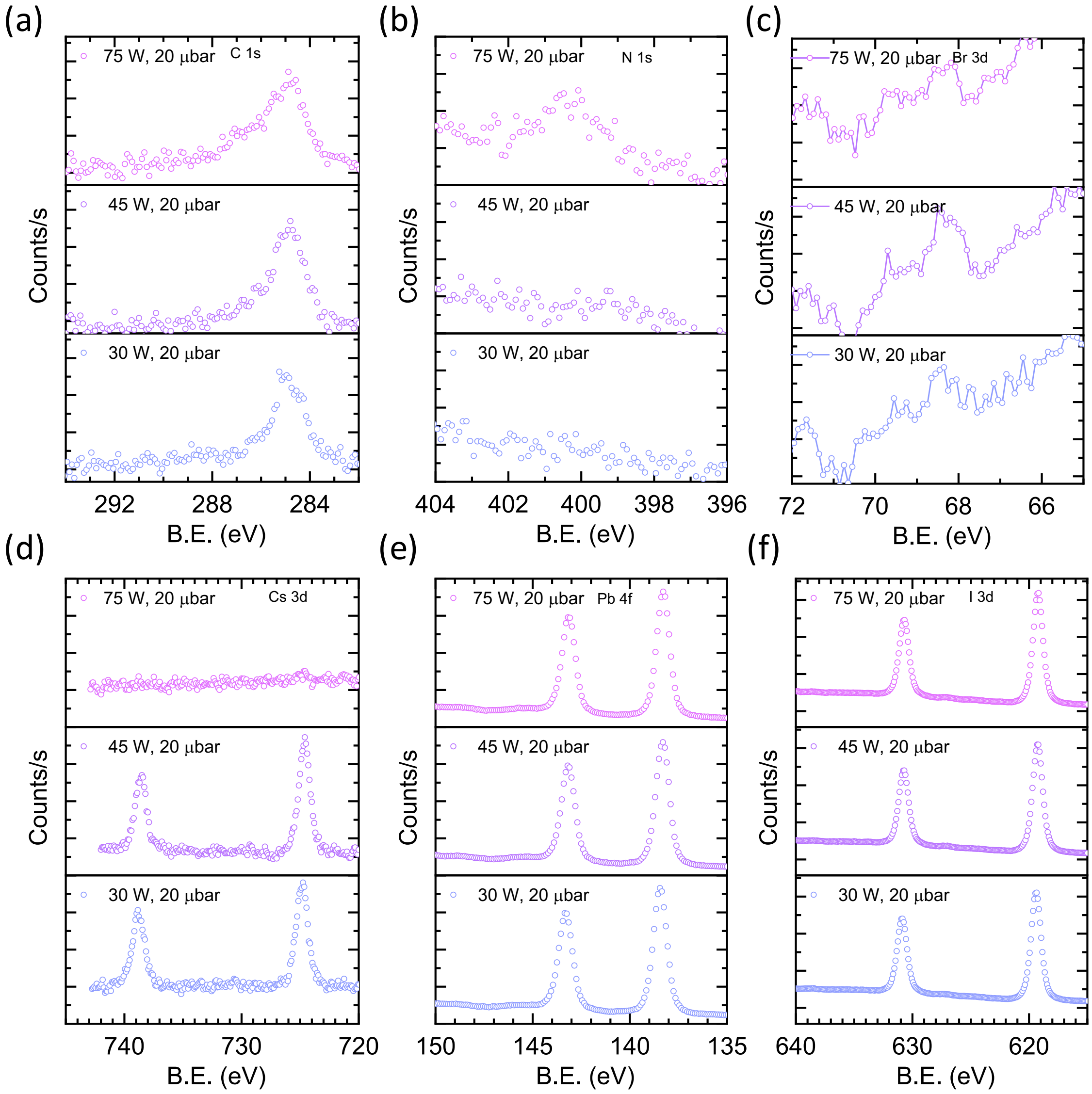

Figure S10. (a) C 1s, (b) N 1s, (c) Br 3d, (d) Cs 3d, (e) Pb 4f, and (f) I 3d core level spectra in sputtered thin films at different powers and fixed pressure.

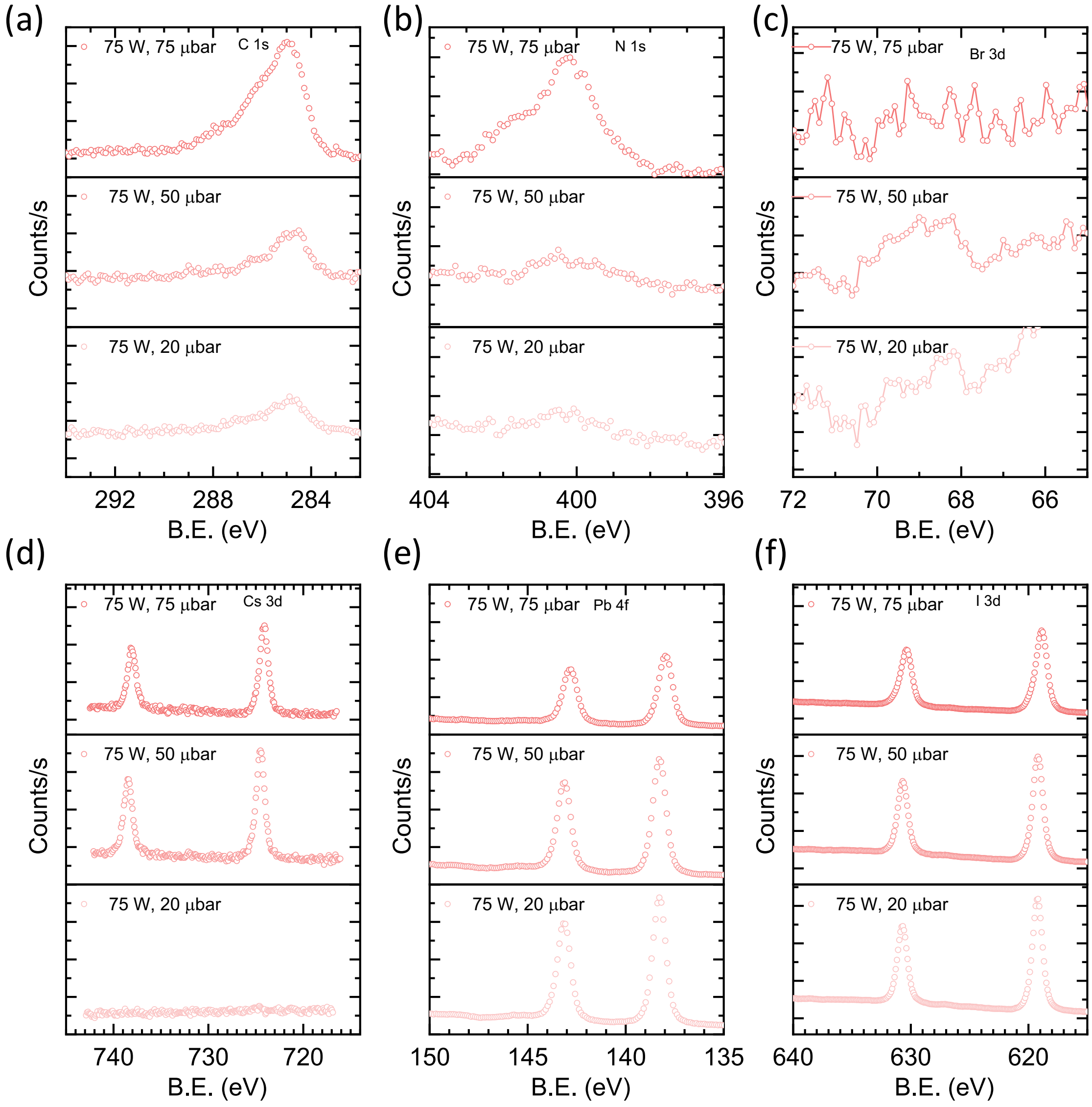

Figure S11. (a) C 1s, (b) N 1s, (c) Br 3d, (d) Cs 3d, (e) Pb 4f, and (f) I 3d core level spectra in sputtered thin films at different pressures and fixed power.

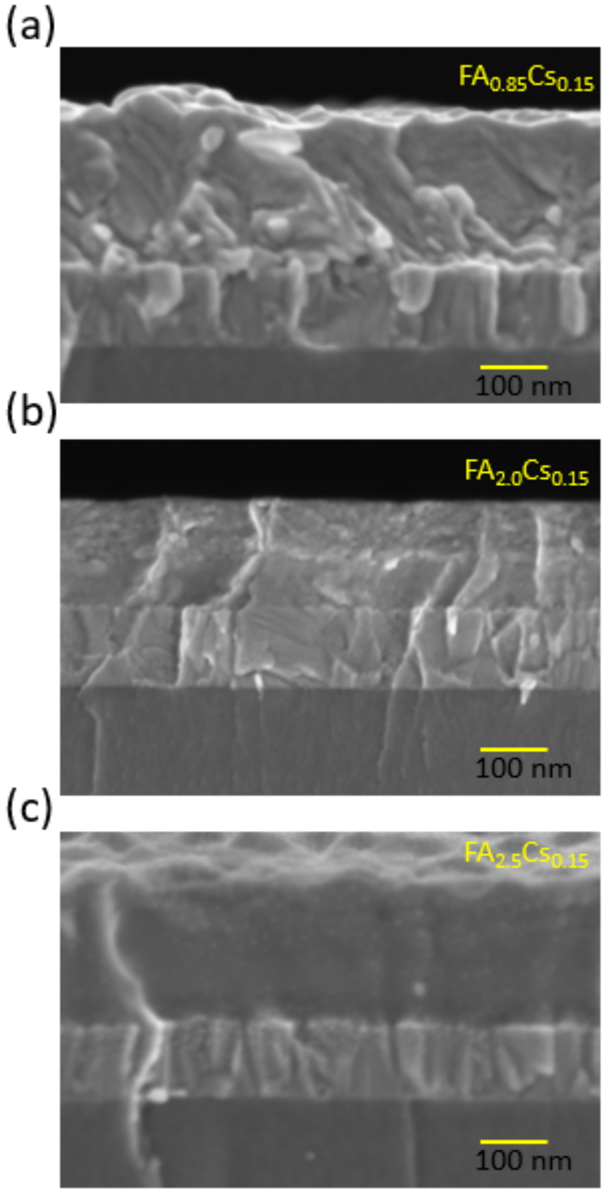

Figure S12. Cross-section scanning electron microscopy (SEM) images of (a) $FA_{0.85}Cs_{0.15}$, (b) $FA_{2.0}Cs_{0.15}$, and (c) $FA_{2.5}Cs_{0.15}$ composition-based sputtered thin films.

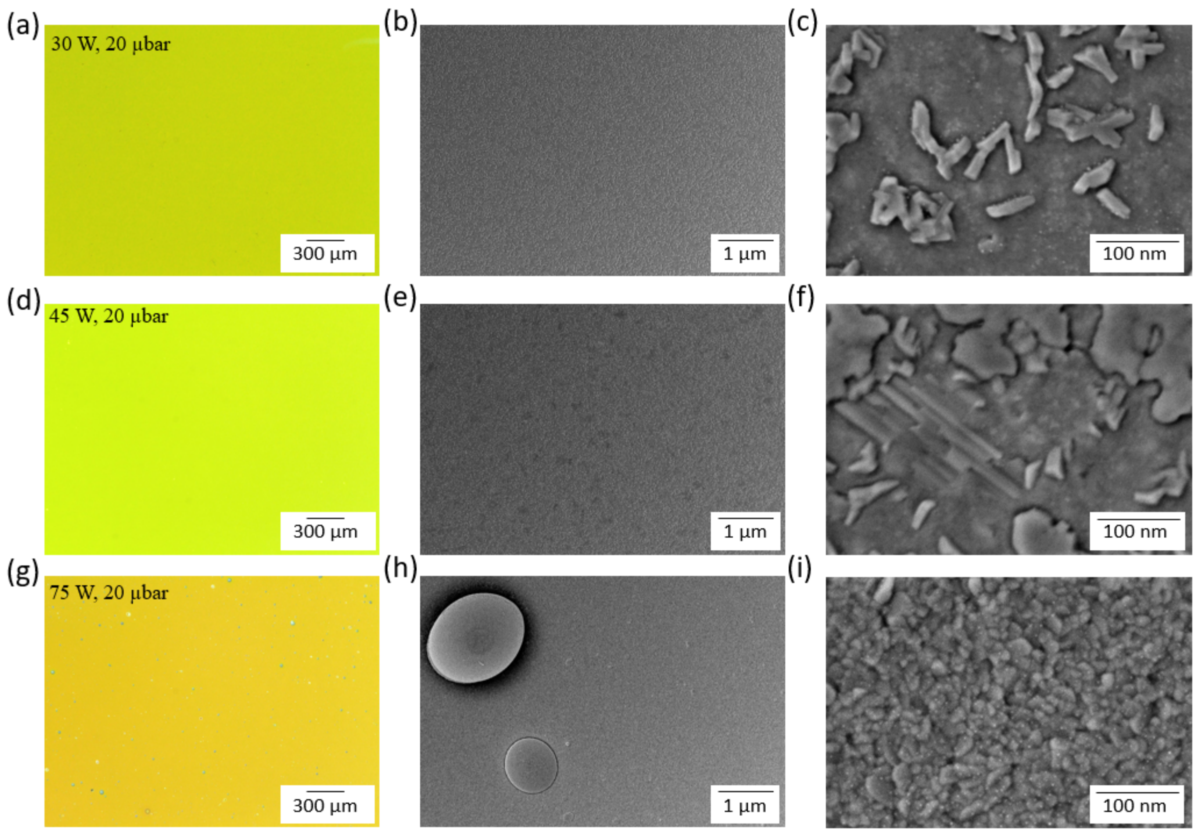

Figure S13. Optical and scanning electron microscopy (SEM) images of sputtered thin films at different deposition powers (a)-(c) 30 W, (d)-(f) 45 W, and (g)-(i) 75 W, where pressure was kept fixed at 20 µbar.

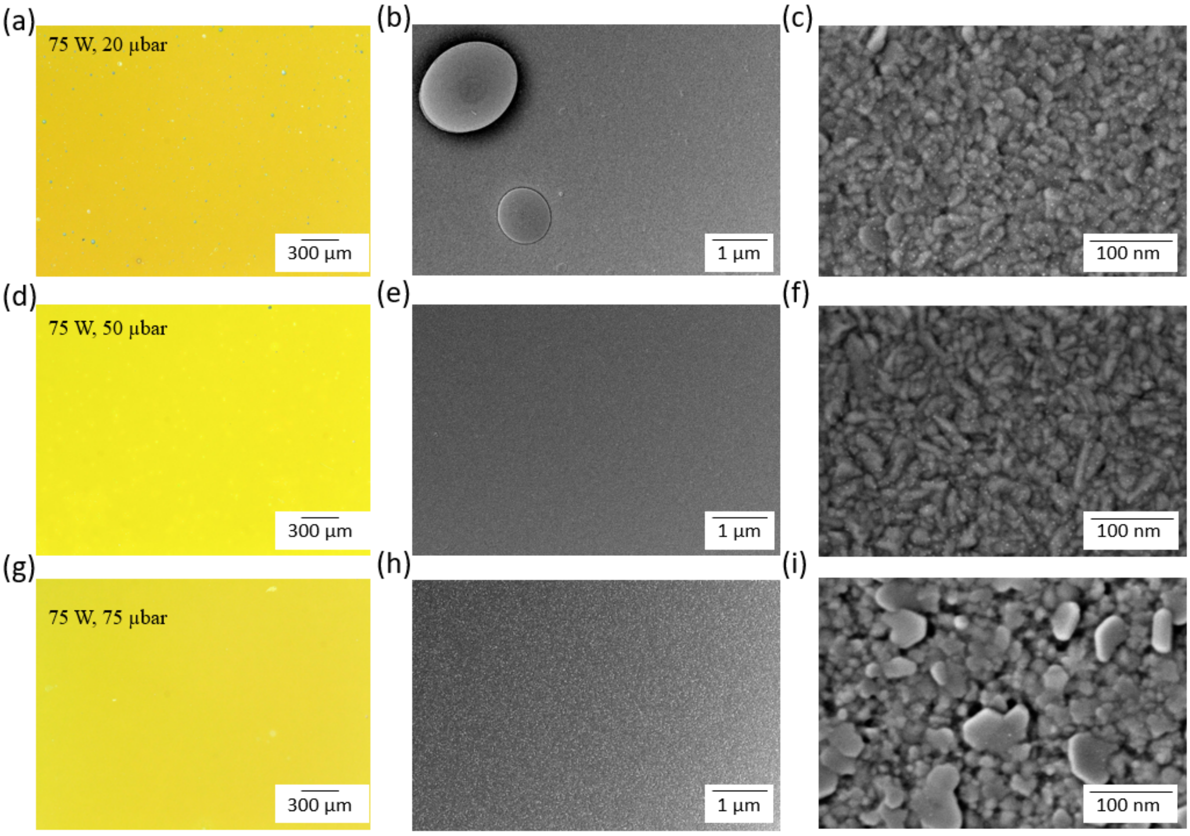

Figure S14. Optical and scanning electron microscopy (SEM) images of sputtered thin films at different deposition pressures (a)-(c) 20 µbar, (d)-(f) 50 µbar, and (g)-(i) 75 µbar, where power was kept fixed at 75 W.

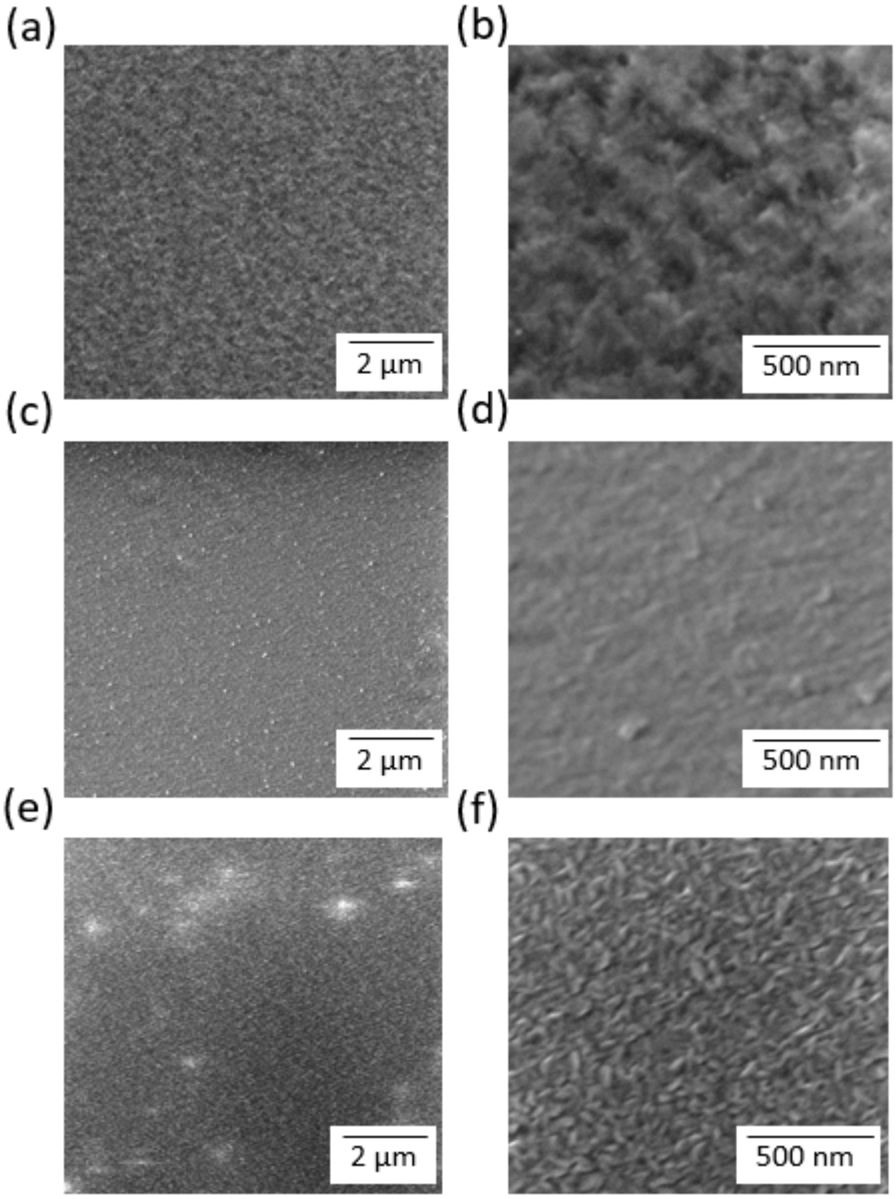

Figure S15. Scanning electron microscopy (SEM) images of (a) & (b) $Cs_{1.0}$, (c) & (d) $Cs_{1.5}$, and (e) & (f) $Cs_{2.0}$ composition-based sputtered thin films.

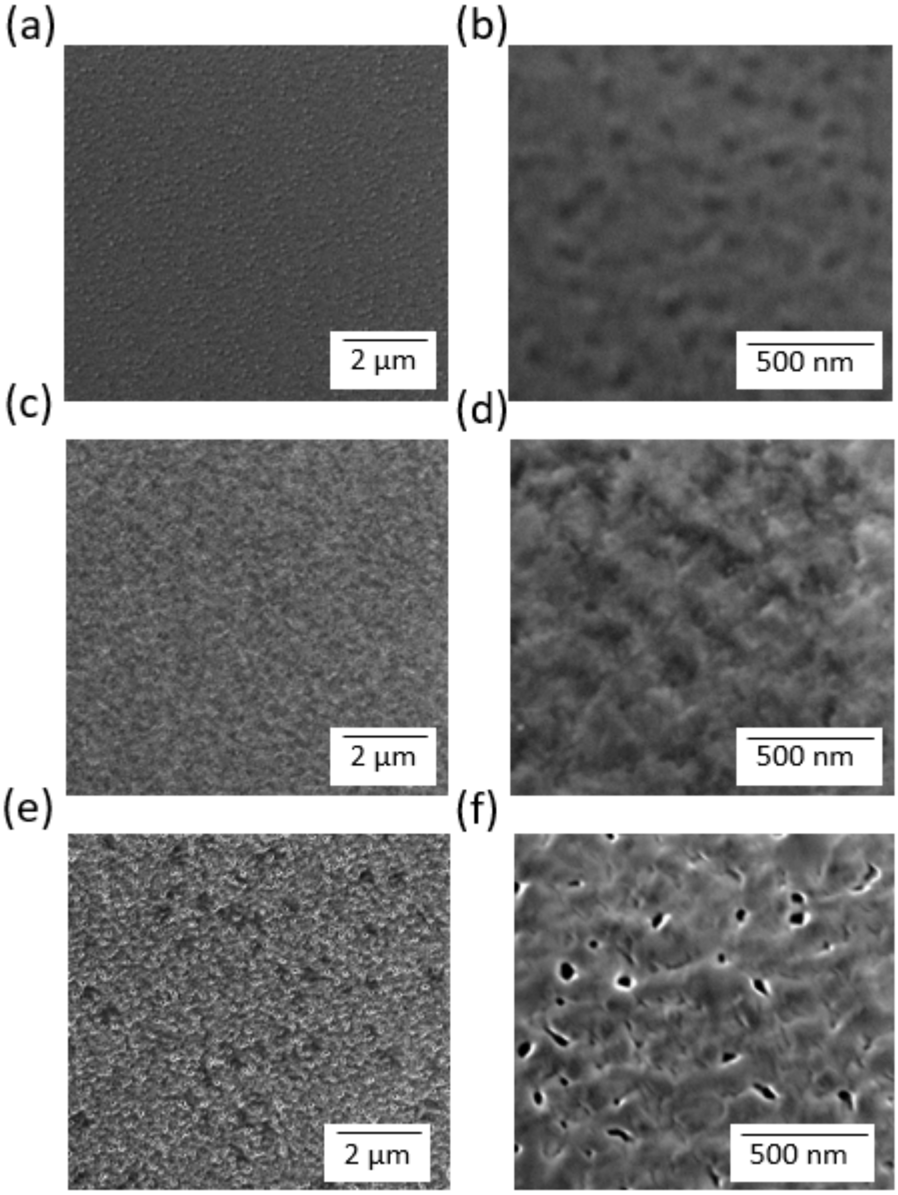

Figure S16. Scanning electron microscopy (SEM) images of sputtered thin films of $Cs_{1.0}$ composition at different deposition powers (a) & (b) 30 W, (c) & (d) 45 W, and (e) & (f) 75 W, where pressure was kept fixed at 20 µbar.

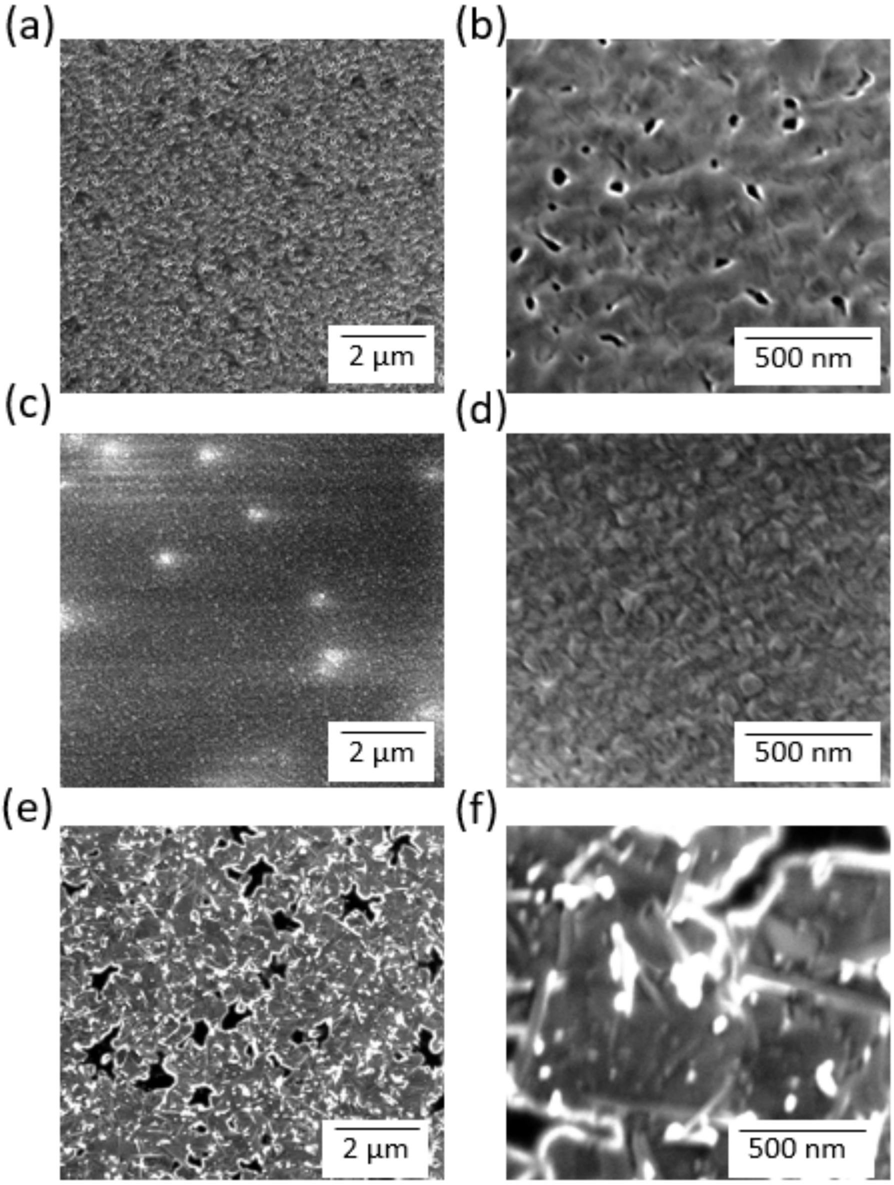

Figure S17. Scanning electron microscopy (SEM) images of sputtered thin films of $Cs_{1.0}$ composition at different deposition pressures (a) & (b) 20 µbar, (c) & (d) 45 µbar, and (e) & (f) 75 µbar, where power was kept fixed at 75 W.

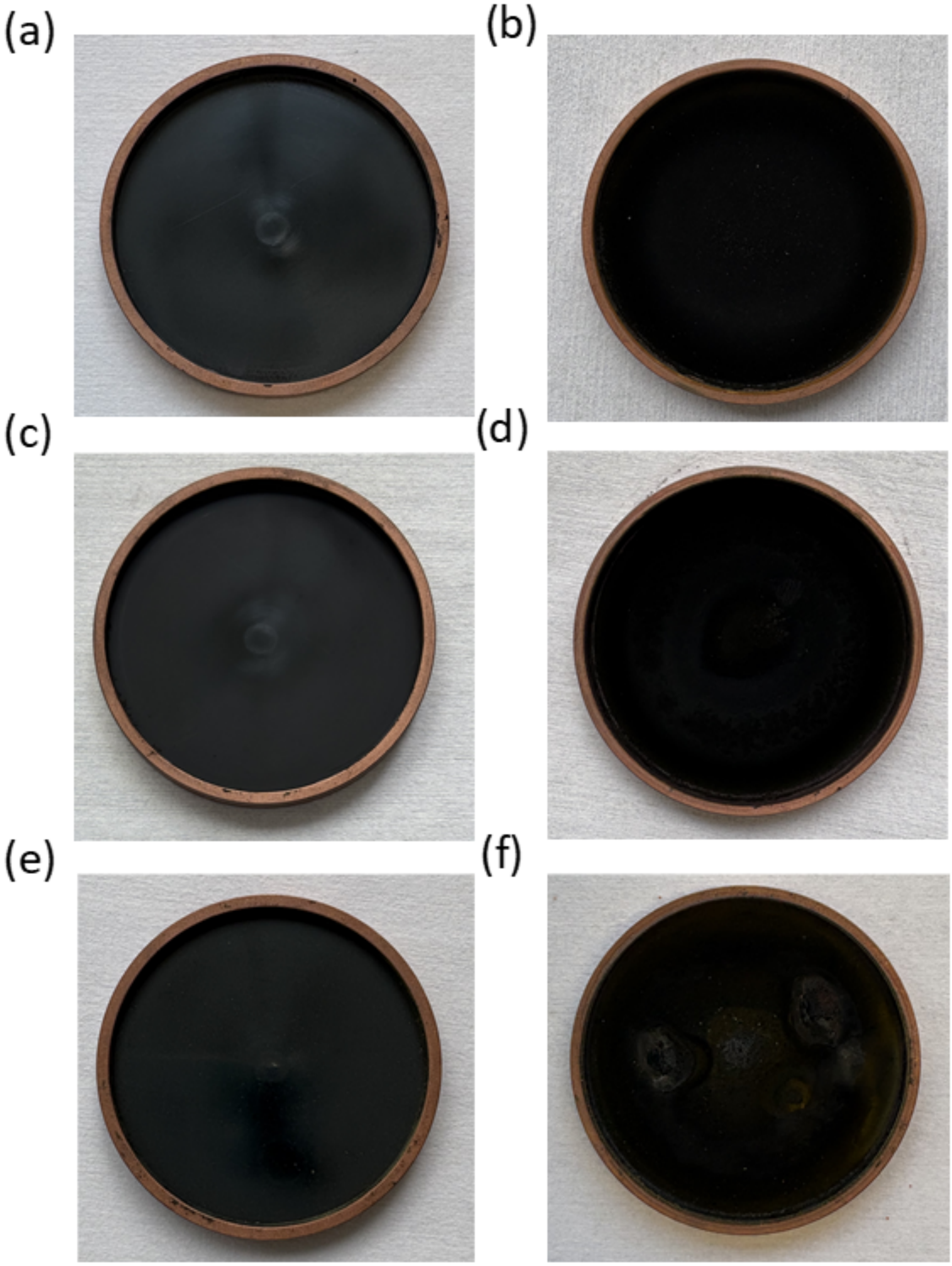

Figure S18. Pictures of target with $FA_{0.85}Cs_{0.15}$ composition at different power (a) & (b) 30 W, (c) & (d) 45 W, and (e) & (f) 75 W, argon gas pressure is fixed at 20 μbar, left panel (before sputtering) and right panel (after sputtering).

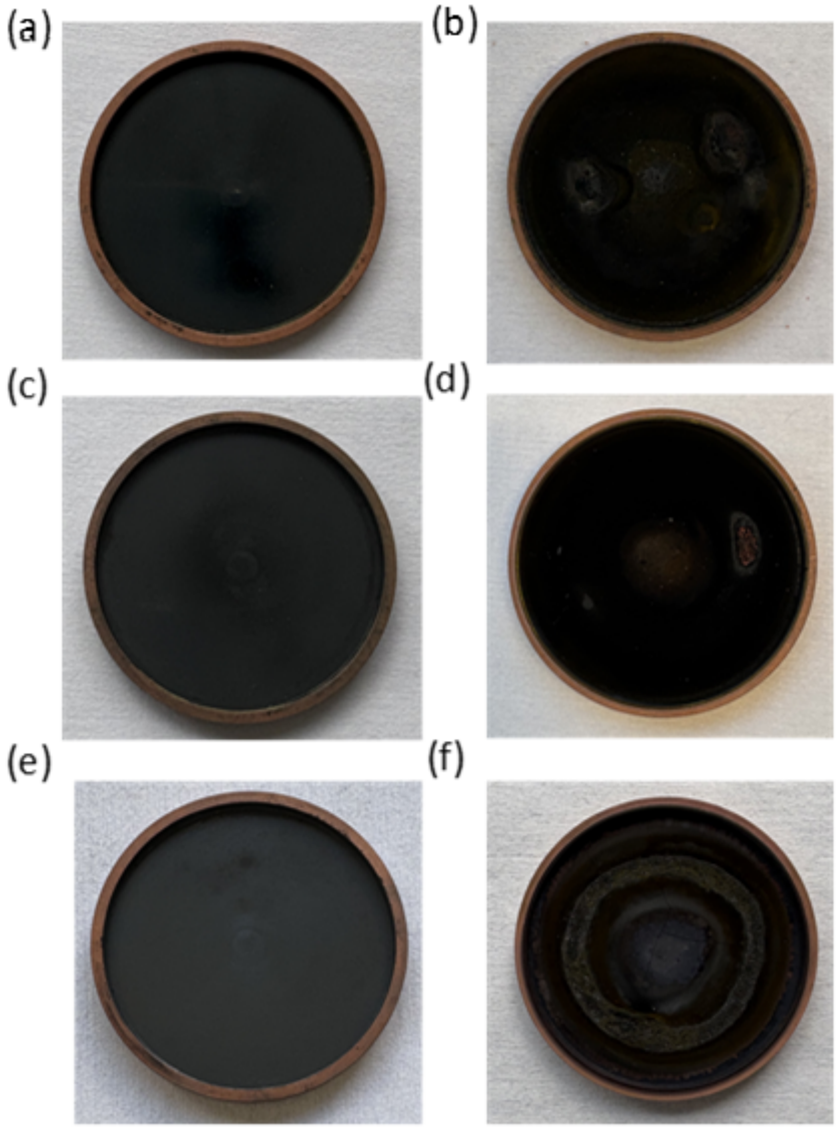

Figure S19. Pictures of target with $FA_{0.85}Cs_{0.15}$ composition at different argon gas pressure (a) & (b) 20 µbar, (c) & (d) 50 µbar, and (e) & (f) 75 µbar, applied power is fixed at 75 W, left panel (before sputtering) and right panel (after sputtering).

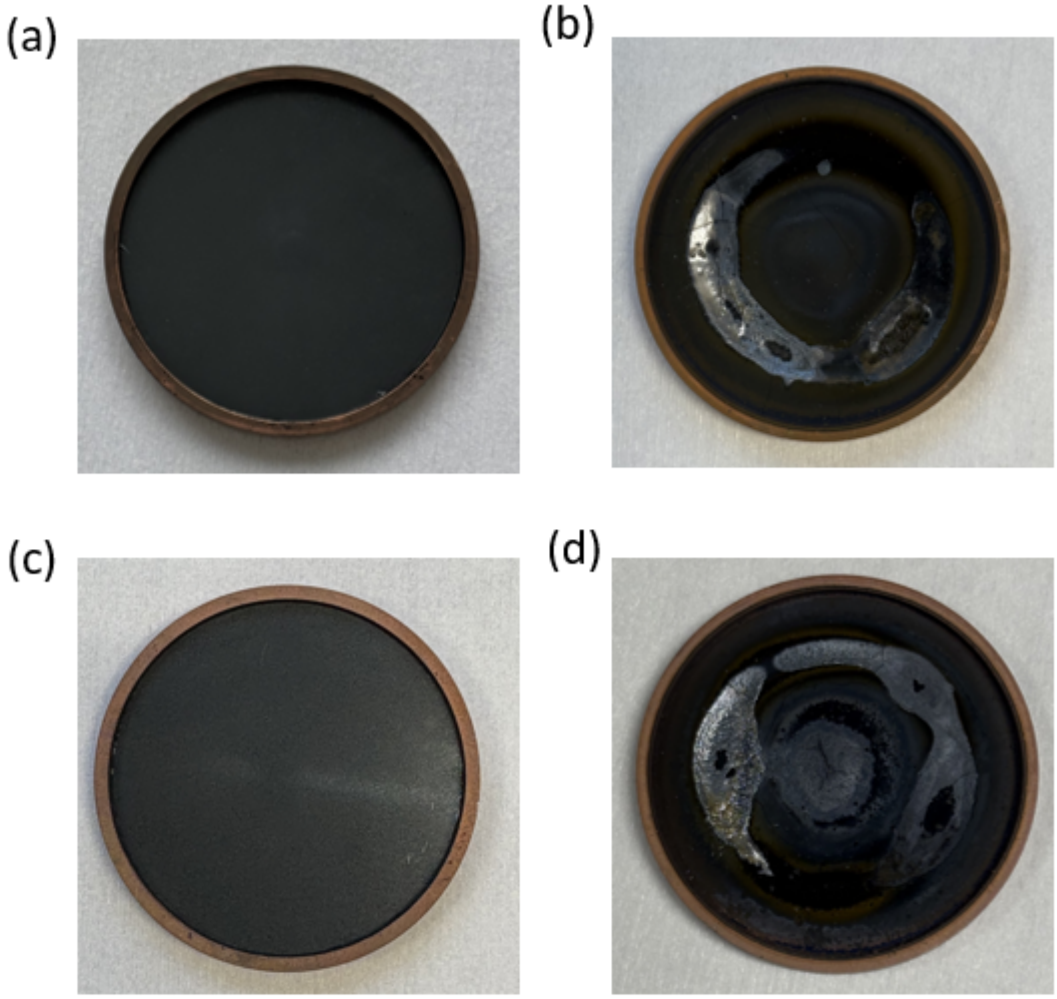

Figure S20. Pictures of target with $FA_{0.85}Cs_{0.15}$ composition at different argon gas pressure (a) & (b) 30 μbar, and (c) & (d) 95 μbar, applied power is fixed at 75 W, left panel (before sputtering) and right panel (after sputtering).

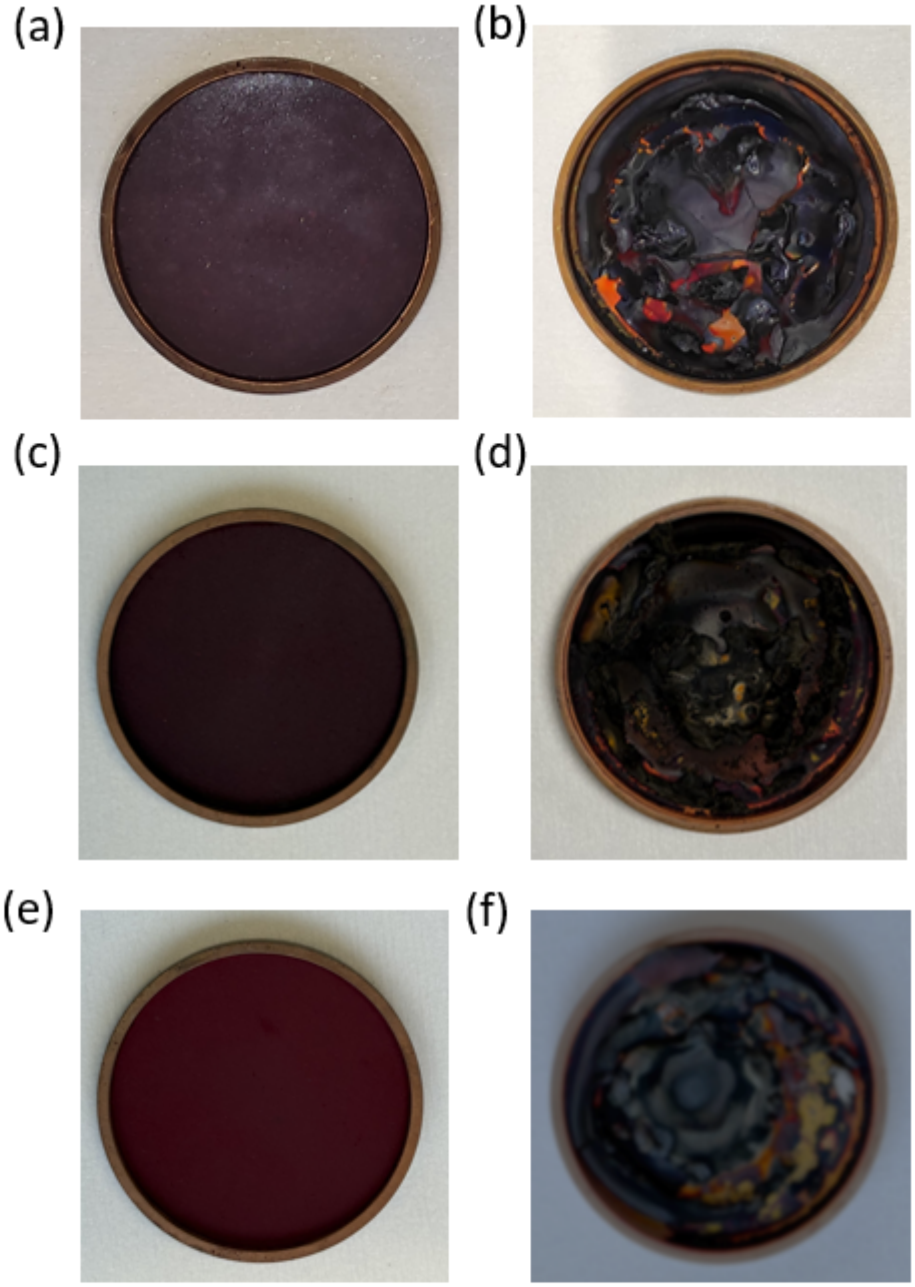

Figure S21. Pictures of target with different compositions (a) & (b) $Cs_{1.0}$, (c) & (d) $Cs_{1.5}$, and (e) & (f) $Cs_{2.0}$, left panel (before sputtering) and right panel (after sputtering).

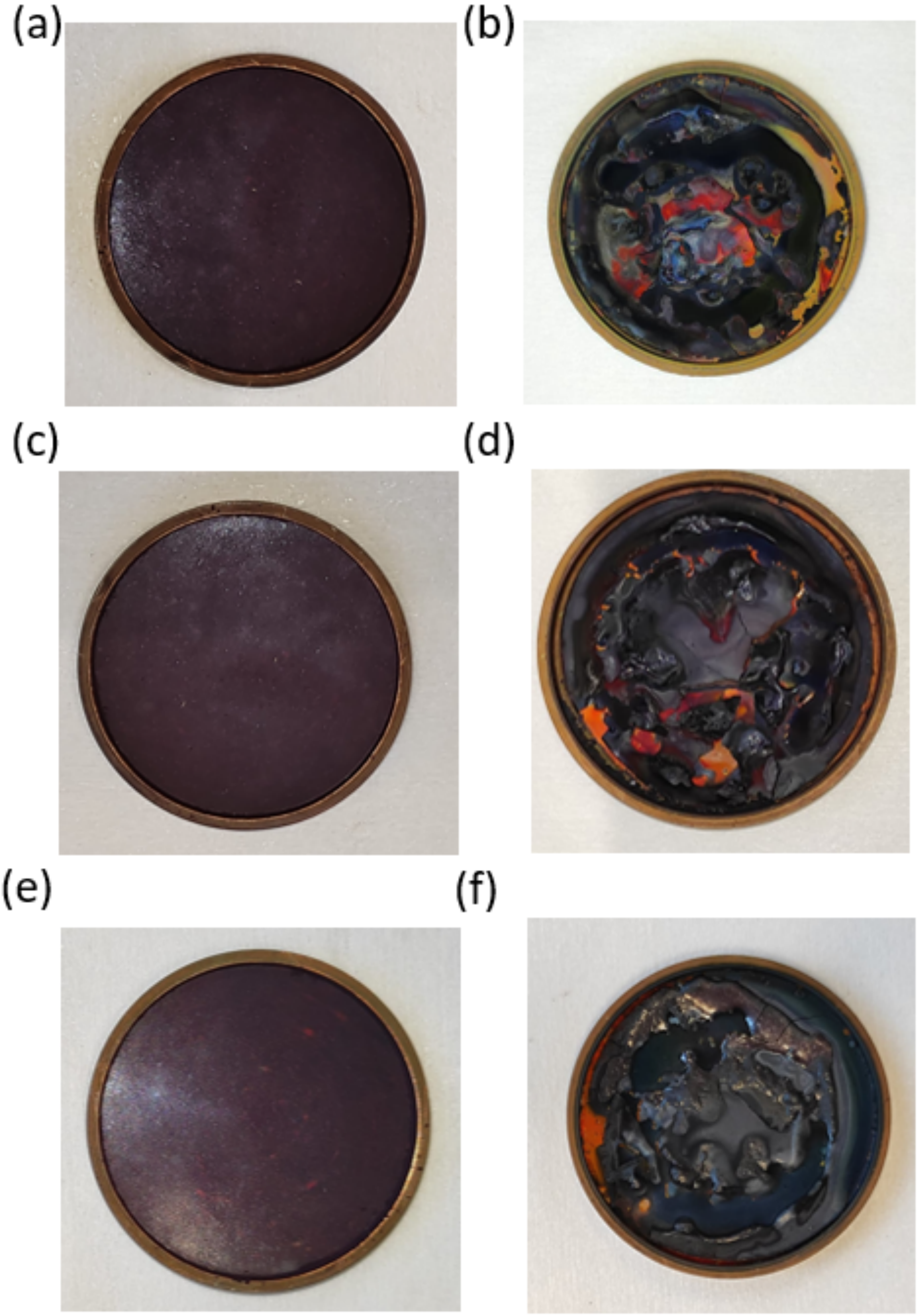

Figure S22. Pictures of target with $Cs_{1.0}$ composition at different power (a) & (b) 30 W, (c) & (d) 45 W, and (e) & (f) 75 W, argon gas pressure is fixed at 20 µbar, left panel (before sputtering) and right panel (after sputtering).

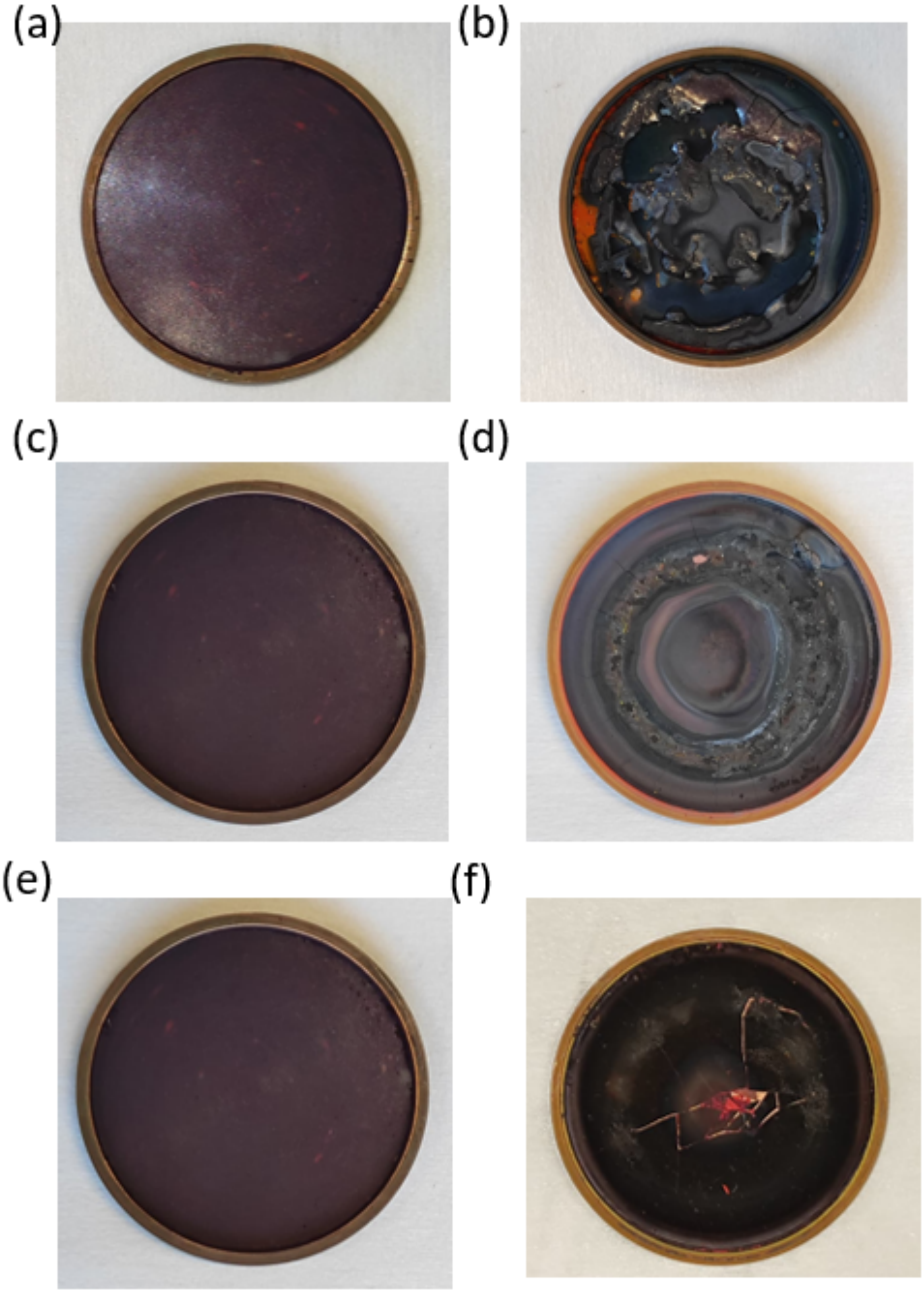

Figure S23. Pictures of target with $Cs_{1.0}$ composition at different argon gas pressure (a) & (b) 20 μbar, (c) & (d) 50 μbar, and (e) & (f) 75 μbar, applied power is fixed at 75 W, left panel (before sputtering) and right panel (after sputtering).

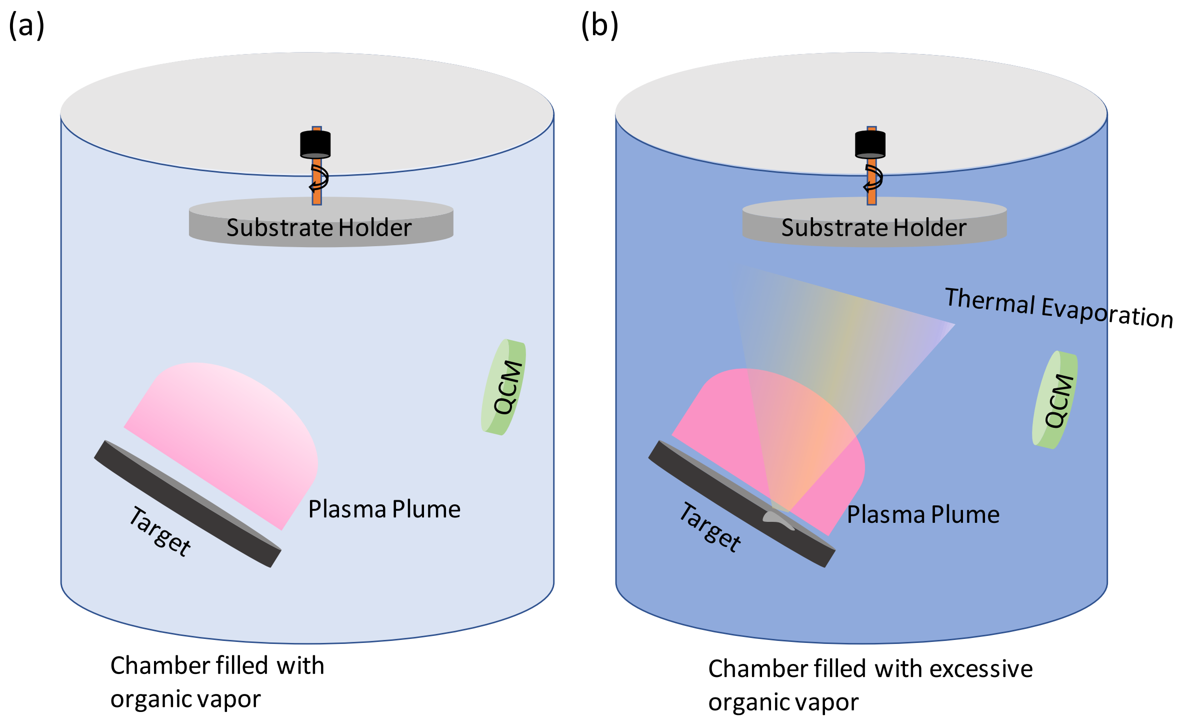

Figure S24. Sputtering mechanism in case of (a) low RF Power (30 W) and (b) high RF power (75 W) for stoichiometric target composition. Based on the results of the sputtered thin films, we hypothesize that at low power, sputtering occurs gently and the organic cation continuously escapes from the target. When we exceed a specific power limit, the target becomes very hot and starts cracking, possibly accompanied by thermal evaporation. At the high-power regime, the deposition rate is uncontrollable due to target cracking, and the resultant film is comparatively less organic-deficient because the chamber is completely filled with excess organic vapor.

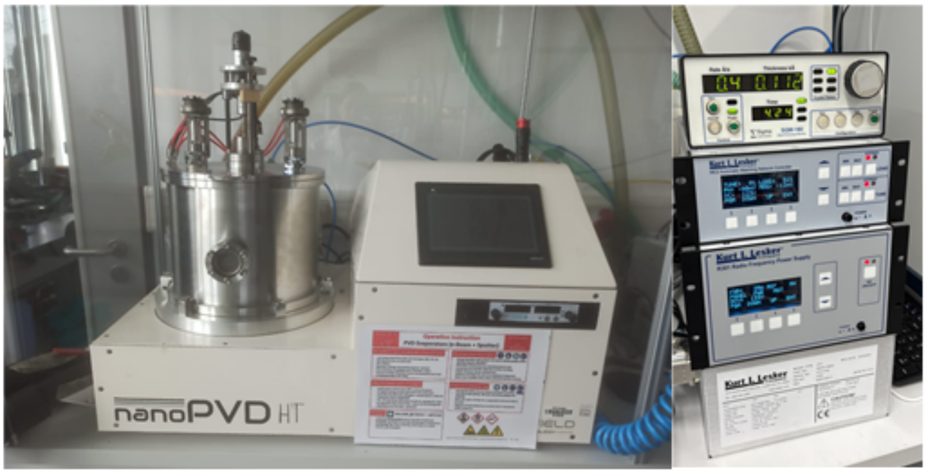

Figure S25. A Nano PVD tabletop sputtering machine is used for depositing thin films.
